# Vaccine Efficacy of ALVAC-HIV and Bivalent Subtype C gp120/MF59 in Adults

**DOI:** 10.1056/NEJMoa2031499

**Published:** 2021-03-25

**Authors:** Glenda E Gray, Linda-Gail Bekker, Fatima Laher, Mookho Malahleha, Mary Allen, Zoe Moodie, Nicole Grunenberg, Yunda Huang, Doug Grove, Brittany Prigmore, Jia J. Kee, David Benkeser, John Hural, Craig Innes, Erica Lazarus, Graeme Meintjes, Nivashnee Naicker, Dishiki Kalonji, Maphoshane Nchabeleng, Modulakgotla Sebe, Nishanta Singh, Philip Kotze, Sheetal Kassim, Thozama Dubula, Vimla Naicker, William Brumskine, Cleon N Ncayiya, Amy M Ward, Nigel Garrett, Girisha Kistnasami, Zakir Gaffoor, Pearl Selepe, Philisiwe B Makhoba, Matsontso P Mathebula, Pamela Mda, Tania Adonis, Katlego S Mapetla, Bontle Modibedi, Tricia Philip, Gladys Kobane, Carter Bentley, Shelly Ramirez, Simbarashe Takuva, Megan Jones, Mpho Sikhosana, Millicent Atujuna, Michele Andrasik, Nima S. Hejazi, Adrian Puren, Lubbe Wiesner, Sanjay Phogat, Carlos Diaz Granados, Marguerite Koutsoukos, Olivier Van Der Meeren, Susan W. Barnett, Niranjan Kanesa-thasan, James G. Kublin, M. Juliana McElrath, Peter B. Gilbert, Holly Janes, Lawrence Corey

**Affiliations:** 1 **GEG:** SAMRC. PHRU, HVTN, Vaccine and Infectious Disease Division, Fred Hutchinson Cancer Research Center, Seattle, Washington, United States of America; 2 **FL, EL, ST, BM, TP:** Perinatal HIV Research Unit, Faculty of Health Sciences, University of the Witwatersrand, Johannesburg, South Africa; 3 **ZM, HJ, YH, DG, BP, JJK, NG, JGK, MJM, PBG, LC, SR, MJ, ST, CB, MS, MA, JH:** Vaccine and Infectious Disease Division, Fred Hutchinson Cancer Research Center, Seattle, Washington, United States of America; 4 **LGB, SK, MA, CNN:** Desmond Tutu HIV Centre, University of Cape Town, Cape Town, South Africa; 5 **MA** Vaccine Research Program, Division of AIDS, National Institute of Allergy and Infectious Diseases, National Institutes of Health, Bethesda, Maryland, United States of America; 6 **MM, KSM** Setshaba Research Centre, Soshanguve, South Africa; 7 **DB:** Department of Biostatistics and Bioinformatics, Rollins School of Public Health, Emory University, Atlanta, Georgia, United States of America; 8 **MN, MPM:** Mecru Clinical Research Unit (MeCRU), Sefako Mkgatho Health Sciences University, GaRankuwa, South Africa; 9 **TD, PM:** Nelson Mandela Academic Clinical Research Unit (NeMACRU) and Department of Internal Medicine and Pharmacology, Walter Sisulu University, Mthatha, South Africa; 10 **GM, AMW:** Department of Medicine, Wellcome Centre for Infectious Diseases Research in Africa, and Institute of Infectious Disease and Molecular Medicine, University of Cape Town, South Africa; 11 **ST:** School of Health Systems and Public Health, Faculty of Health Sciences, University of Pretoria, South Africa; 12 **SWB:** GSK Vaccines, Cambridge, MA, USA;; 13 **LW:** Division of Clinical Pharmacology, Department of Medicine, University of Cape Town, Cape Town, South Africa; 14 **NS, DK, GK, ZG:** South African Medical Research Council, Durban, South Africa; 15 **NKT:** GSK Vaccines, Rockville MD USA; 16 **CDG, SP:** Sanofi Pasteur, Swiftwater, Pennsylvania, United States of America; 17 **SP:Current affiliation:** GSK, Siena, Italy; 18 **MK:** GSK, Wavre, Belgium; 19 **OVDM:** GSK, Rixensart, Belgium; 20 **AP:** National Institute for Communicable Diseases, National Health Laboratory Service, Johannesburg, South Africa; 21 **MS, GK, CI, PS, TA, WB:** Aurum Institute, South Africa; 22 **NSH:** Graduate Group in Biostatistics, University of California, Berkeley, Center for Computational Biology, University of California, Berkeley, CA, USA; 23 **NN, NG:** Centre for the AIDS Programme of Research in South Africa (CAPRISA), University of KwaZulu–Natal, Durban, South Africa; 24 **PK, PBM:** Qhakaza Mbokodo Research Clinic, Ladysmith, South Africa

## Abstract

**BACKGROUND:**

A safe, effective vaccine is essential to end HIV. A canarypox/protein HIV vaccine regimen showed modest efficacy at reducing infection in Thailand. An analogous regimen using HIV-1 subtype C virus demonstrated potent humoral and cellular responses in a Phase 1/2a trial and triggered a Phase 2b/3 double-blinded trial to assess the safety and efficacy of this regimen in South Africa.

**METHODS:**

We enrolled and randomized 5,404 healthy, HIV-uninfected 18-35-year olds at 14 sites to vaccine (2,704 participants) or placebo (2,700 participants) between 26 October 2016 and 21 June 2019. The vaccine regimen consisted of two ALVAC-HIV (vCP2438) (expressing HIV-1 subtype C *env,* clade B *gp41*, *gag* and *pro)* immunizations at months 0 and 1, with booster immunizations of ALVAC-HIV plus bivalent subtype C gp120 protein/MF59 adjuvant at months 3, 6, 12 and 18. Efficacy was evaluated by HIV testing every 3 months.

**RESULTS:**

In January 2020, pre-specified non-efficacy criteria were met at an interim analysis; further vaccinations were subsequently halted. The vaccines were safe and well-tolerated in the study population (median age 24, 70% female-sex-at-birth). Over the primary 24-month follow-up, there were 133 infections among placebo recipients and 138 among vaccinees (hazard ratio = 1.02; 95%CI, 0.81-1.30; P=0.84). Pre-specified subgroup analyses demonstrated no difference in efficacy by sex or when restricting to follow-up post-4^th^ vaccination, and no difference amongst female-sex-at-birth by age, BMI, prevalent STIs, behavioral risk score or region.

**CONCLUSIONS:**

The ALVAC/gp120 regimen did not prevent HIV infection in South Africans despite prior evidence of immunogenicity.

ClinicalTrials.gov (NCT02968849)

## Introduction

Most of the 75.7 million people infected with HIV are from sub-Saharan Africa, where HIV-1 subtype C is prevalent.^1^ South Africa (SA) bears a disproportionately large HIV burden with approximately 7.9 million persons living with HIV, highlighting the urgent need for a vaccine.^2^

In 2010, following the announcement that the RV144 HIV vaccine trial demonstrated 31% efficacy in a community-based trial in Thailand,^3^ the Pox-Protein Public-Private Partnership (P5) was established. The P5 developed an analogous regimen using HIV-1 subtype C sub-Saharan African strains, including a transmitted-founder isolate.^4^ This approach, utilizing a recombinant canarypox vector containing a subtype C envelope (*env*) ALVAC-HIV (vCP2438) and MF59-adjuvanted subtype C bivalent gp120 protein vaccine, was safe and induced strong humoral and cellular immune responses.^5^ We investigated the safety and efficacy of this vaccine regimen against HIV-1 acquisition in SA.

## Methods

### TRIAL DESIGN

HIV Vaccine Trials Network (HVTN) 702 was a Phase 2b/3 randomized double-blind placebo-controlled trial of ALVAC-HIV (vCP2438) and MF59-adjuvanted bivalent subtype C gp120. Participants were randomized 1:1 to vaccine or placebo, stratified by sex-at-birth and site, with centrally generated randomization by the Statistical Center for HIV/AIDS Research and Prevention (SCHARP). The trial was designed to evaluate vaccine efficacy **(**VE) to prevent HIV infection within 24 months of enrollment (i.e., VE(0-24)) with formal monitoring for potential harm, non-efficacy, and high efficacy, with potential to extend follow-up to 36 months.^6^

### TRIAL POPULATION

Eligible participants were consenting, healthy, HIV-uninfected 18 to 35-year old adults at 15 community sites in SA. We aimed to enroll 60-75% persons assigned female-sex-at-birth (females hereafter). Females of reproductive potential were required to use contraception until 3 months post-final vaccination; pregnant/breast-feeding females were excluded.

### INTERVENTION

The vaccines were ALVAC-HIV (vCP2438) and MF59-adjuvanted bivalent subtype C gp120. ALVAC-HIV (vCP2438) (nominal dose of 10⁷ 50% cell culture infectious dose) expressed the HIV-1 *env* gp120 of the subtype C ZM96.C strain with the gp41 transmembrane sequence, *gag* and *protease* from the subtype B LAI strain. Bivalent subtype C gp120 was a combination of 100 mcg each of the HIV-1 subtype C gp120 of the TV1.C and 1086.C strains. Placebo was Sodium Chloride for Injection 0.9%, USP.

Participants received either ALVAC-HIV at Months 0 and 1 followed by 4 administrations of ALVAC-HIV plus bivalent subtype C gp120/MF59 at Months 3, 6, 12 and 18, or placebo by intramuscular injection. ALVAC-HIV or placebo was administered in the left deltoid while bivalent subtype C gp120/MF59 or placebo was administered in the right deltoid.

### OUTCOME EVALUATION

Study visits were scheduled at Months 0, 1, 3, 6, 6.5, 12, 12.5, 15, 18, 18.5, 21 and every three months thereafter up to Month 36 with vaccine safety (VE) evaluated (see Supplementary Materials). Physical examination, HIV risk reduction counseling, pregnancy assessment, social impact assessment, adverse event (AE) monitoring and collection of concomitant medications were performed at every visit. HIV testing with counseling occurred every 3 months, sexually transmitted infection (STI) testing was done every 6 months and a behavioral risk questionnaire was performed at screening, months 6.5, 12, 24, and 36. Access to free pre-exposure prophylaxis and post-exposure prophylaxis (PrEP/PEP) was provided. Vaccinations were prohibited during pregnancy and breastfeeding.

### LABORATORY METHODS

HIV testing was done at study sites to avoid potential unblinding of treatment. HIV infection was confirmed by detection of HIV nucleic acid and HIV-1 RNA viral loads were measured post-diagnosis. An independent adjudication committee reviewed HIV diagnostic test results from specimens collected on at least two dates and made the primary determination of infection status and timing. For monitoring PrEP/PEP use, dried blood spot (DBS) samples were collected on all participants seen at sites on a given day each month to measure tenofovir diphosphate (TFV-DP).

### TRIAL OVERSIGHT

The trial was designed by investigators, the P5 and collaborators. All data were collected and analyzed by SCHARP. All authors had access to data and critically reviewed and approved the manuscript. The first draft was written by the three lead authors, the statisticians and the senior author. HVTN 702 Study Team is listed in **Table S1**.

### ETHICAL CONSIDERATIONS

All participants provided written informed consent in their preferred language before screening. The research ethics committees of the Universities of the Witwatersrand, Cape Town, KwaZulu-Natal, Sefako Makgatho University and the South African Medical Research Council approved the trial. The trial was overseen by the NIAID HIV Vaccine Data and Safety Monitoring Board (DSMB) and registered with the South African National Clinical Trials Register (SANCTR number: DOH-27-0916-5327) and ClinicalTrials.gov (NCT02968849).

### STATISTICAL ANALYSIS

The primary efficacy outcome, VE(0-24), was measured as 1 minus the hazard ratio for HIV-1 infection, estimated using a sex-stratified Cox proportional-hazards model and tested using a sex-stratified log-rank test. VE was also measured using a ratio of cumulative incidences of HIV-1 infection, vaccine vs. placebo, and estimated using transformed Nelson-Aalen cumulative hazard functions. Secondary analyses evaluated VE from Month 6.5 to 24 (VE(6.5-24)), starting two weeks post-4^th^ vaccination, and VE from Month 0 to 36 (VE(0-36)). Follow-up time was the number of days from randomization to HIV-1 diagnosis; or for participants without HIV-1 infection diagnosis, from randomization to the last HIV-1 negative test or to the end of the Month 24 (or Month 36) visit window, whichever occurred first. Kaplan-Meier plots show the cumulative incidence of time to HIV-1 infection and loss-to-follow-up. Pre-specified baseline variables were evaluated as modifiers of VE by Wald interaction tests, using stratified Cox proportional hazards models with Holm^7^ multiplicity adjustment.

The primary efficacy analysis was based on the modified intention-to-treat (MITT) cohort, defined as all enrolled participants, apart from those diagnosed with HIV-1 infection on the day of enrollment, and analyzed according to the randomized treatment. Secondary efficacy analyses evaluated the Month 6.5 at-risk cohort (MITT participants on-study and HIV-1-negative at Month 6.5, at risk of subsequent HIV-1 infection) and the per-protocol (PP) cohort, defined as participants in the Month 6.5 at-risk cohort who received the first four vaccinations on schedule without error. Safety analyses included all randomized participants who received at least one injection and analyzed according to the treatment received.

The sample size of 5,400 participants provided at least 90% power to reject a null hypothesis of VE(0-24) ≤ 25% if the true VE was 50% or more, based on an assumed 4% placebo group annual HIV-1 incidence (**Tables S2-S3**).

Continuous monitoring for a potential vaccine-induced increased HIV risk began at 12 infections until non-efficacy monitoring commenced at 59 infections, occurring approximately every 6 months through 24 months follow-up of participants. The pre-specified non-efficacy stopping criteria were that the lower limits of 95% confidence intervals (CIs) for both VE(0-24) and VE(6.5-24) lay below 0% and upper limits lay below 40%, for both Cox proportional hazards and cumulative incidence ratio estimation approaches; and that at least 60% of enrolled participants had reached the Month 18.5 visit.

The data presented are on visits and evaluations through February 18, 2020, prior to study unblinding on February 19, 2020.

All P values are two-sided, with P values <0.05 considered statistically significant. Further details are provided in Supplementary Materials.

## Results

### Study population

**Figure 1:**
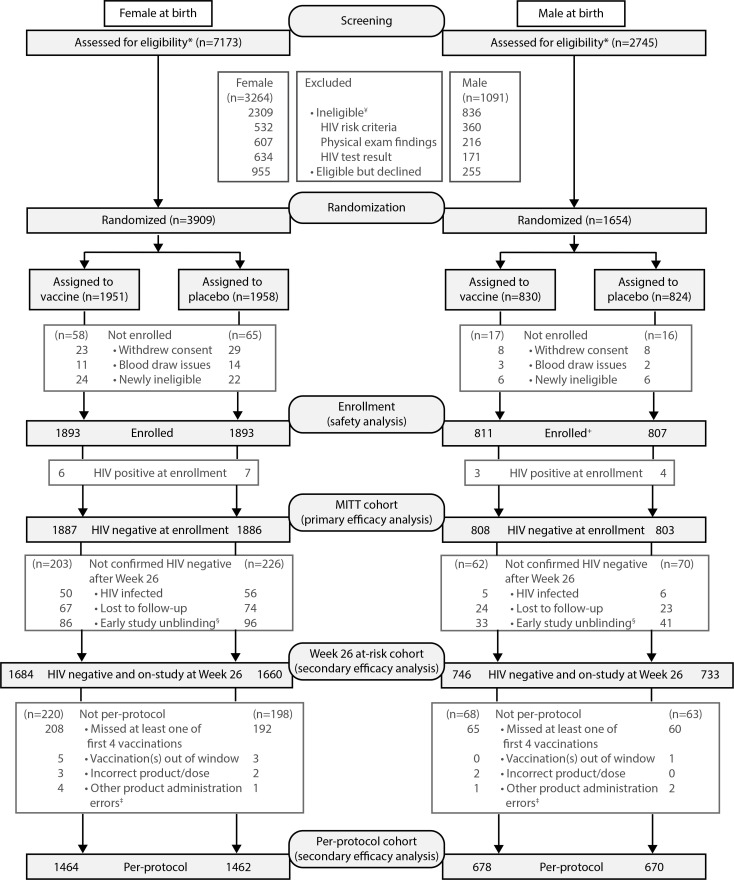
**Screening, randomization, enrollment, and study outcomes.** ^¥^Top 3 reasons for ineligibility shown. ^*^4 participants were screened and found ineligible but sex-at-birth not recorded. ^+^3 participants were randomized and enrolled twice and only data from the first randomization and enrollment is considered. ^§^Participant is not in Month 6.5 at-risk cohort because no HIV diagnostic test was performed after Month 6.5 and prior to study unblinding. ^‡^Other product administration errors include incorrect site of administration, product expired, and other.

Between 26 October 2016 and 21 June 2019, 9,919 individuals were screened and 5,407 enrolled (**Fig. 1**). Of these, three participants were enrolled twice and only data from the first enrollment were used. Of the 5,404 unique participants enrolled, 2,704 were randomized to vaccine and 2,700 to placebo. Baseline demographic, clinical and HIV-1 risk factors were similar between treatment groups (**Table 1**). Overall, 3,786 (70%) participants were female, with 1,115 (29%) aged 18-21 years. Participants assigned male-at-birth (hereafter, male) tended to be older, with 859 (53%) at least 26 years. At enrollment, 1,194/3,389 (35%) females and 264/1,254 (21%) males were diagnosed with STIs. Detailed baseline participant characteristics are in **Tables S4-S9**.

**Table 1:** Baseline characteristics of enrolled participants, by sex-at-birth and treatment assignment.

	Females, n (%)			Males, n (%)		
	Total n=3786	lacebo n=1893	Vaccine n=1893	Total n=1618	Placebo n=807	Vaccine n=811
**Age (years)**						
18-21	1115 (29)	572 (30)	543 (29)	321 (20)	155 (19)	166 (20)
22-25	1420 (38)	697 (37)	723 (38)	438 (27)	219 (27)	219 (27)
26-35	1251 (33)	624 (33)	627 (33)	859 (53)	433 (54)	426 (53)
**BMI**						
<18.5	133 (4)	77 (4)	56 (3)	232 (14)	118 (15)	114 (14)
18.5-24	1406 (37)	681 (36)	725 (38)	1127 (70)	567 (70)	560 (69)
25-29	978 (26)	485 (26)	493 (26)	194 (12)	97 (12)	97 (12)
≥30	1269 (34)	650 (34)	619 (33)	65 (4)	25 (3)	40 (5)
**Gender identity**						
Female	3783 (100)	1891 (100)	1892 (100)	6 (0)	1 (0)	5 (1)
Male	2 (0)	1 (0)	1 (0)	1598 (99)	797 (99)	801 (99)
Transgender female/male	1 (0)	1 (0)	0 (0)	10 (1)	6 (1)	4 (0)
Gender variant	0 (0)	0 (0)	0 (0)	2 (0)	1 (0)	1 (0)
Prefer not to answer	0 (0)	0 (0)	0 (0)	2 (0)	2 (0)	0 (0)
**Condom use (in general)**						
Always	209 (6)	121 (6)	88 (5)	140 (9)	61 (8)	79 (10)
Sometimes	2790 (74)	1379 (73)	1411 (75)	1228 (76)	627 (78)	601 (74)
Never	786 (21)	393 (21)	393 (21)	249 (15)	119 (15)	130 (16)
Exchange of sex for money/gifts*						
Yes	791 (21)	407 (22)	384 (20)	256 (16)	128 (16)	128 (16)
Number of sex acts*						
0-4	1238 (33)	614 (32)	624 (33)	455 (28)	226 (28)	229 (28)
5-10	1373 (36)	671 (35)	702 (37)	609 (38)	299 (37)	310 (38)
≥11	1173 (31)	606 (32)	567 (30)	554 (34)	282 (35)	272 (34)
**Lives with spouse/main partner**						
Yes	530 (14)	291 (15)	239 (13)	278 (17)	134 (17)	144 (18)
**Sexually transmitted infections$**						
Syphilis	44 (1)	23 (1)	21 (1)	26 (2)	16 (3)	10 (2)
Neisseria gonorrhoeae	179 (5)	89 (5)	90 (5)	39 (3)	20 (3)	19 (3)
Chlamydia trachomatis	779 (23)	371 (22)	408 (24)	199 (16)	96 (16)	103 (16)
Trichomonas vaginalis	192 (6)	95 (6)	97 (6)			

* Timeframe for question is the previous 30 days.

$ STI testing was introduced in version 2 of the protocol and so data is not available on 763 participants (397 females and 366 males). Percentages are computed relative to the numbers tested.

The MITT cohort included 5,384 participants (2,689 placebo and 2,695 vaccine) followed for a median of 623 days (interquartile range [IQR]: 427, 819). Month 6.5 at-risk cohort median follow-up was 642 days (IQR: 459, 756) and 644 days in the PP cohort (IQR: 461, 756). Loss-to-follow-up was low (3.9/100 person-years for vaccine group; 3.9/100 person-years for placebo group; **Fig S1**). Protocol adherence was high (**Tables S10, S11**).

### Efficacy

#### HIV-1 infection

**Figure 2:**
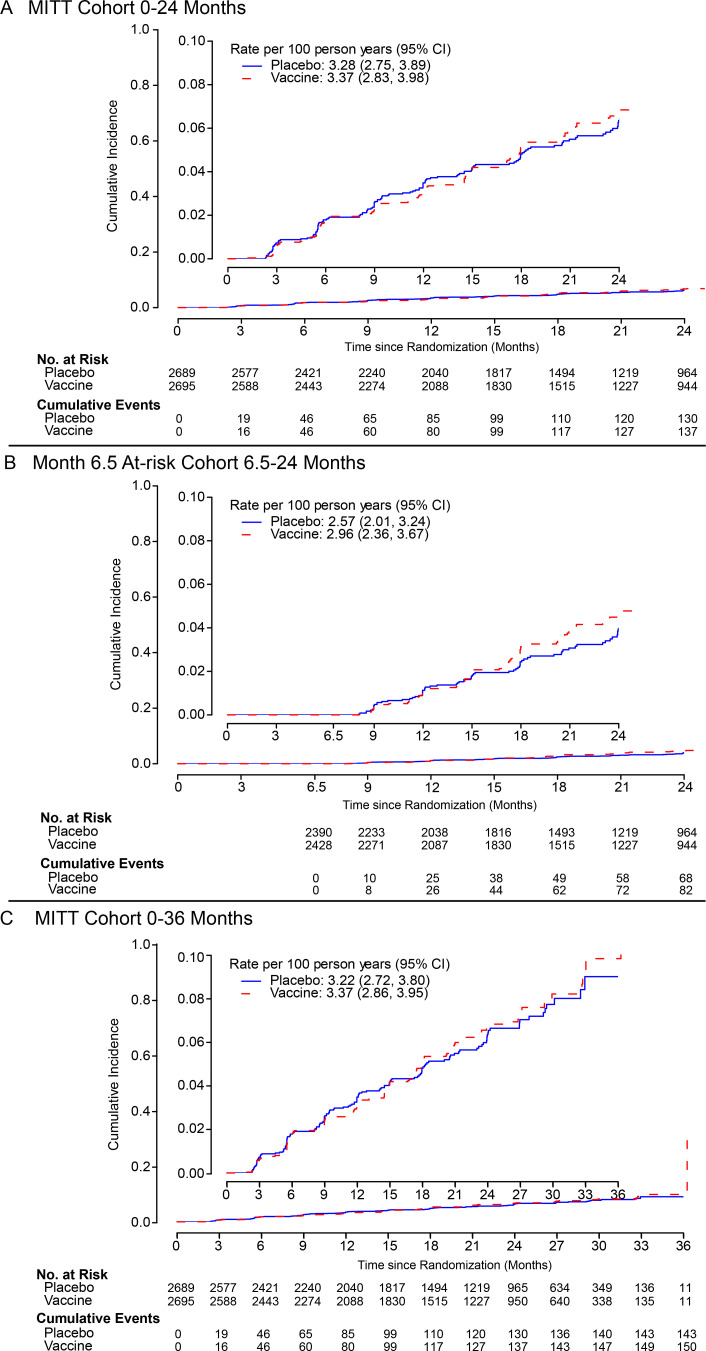
**Cumulative incidence of HIV-1 infection.** A. MITT cohort, 0-24 months. B. Month 6.5 at-risk cohort, 6.5-24 months. C. MITT cohort, 0-36 months. Inset shows the same data on an expanded axis.

**Figure 3:**
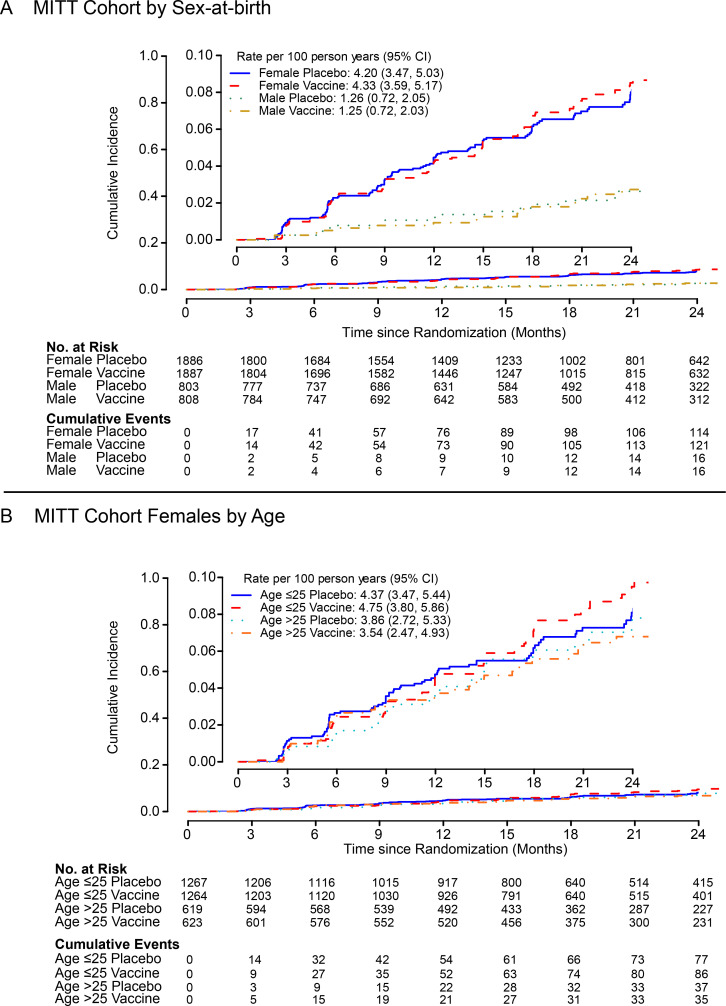
**Cumulative incidence of HIV-1 infection by pre-specified baseline variables.** Cumulative incidence over months 0-24 in the MITT cohort, by sex-at-birth (A) and by age among females (B). Inset shows the same data on an expanded axis.

During the first 24 months of follow-up, 138 HIV-1 infections accrued in the vaccine group and 133 in the placebo group of the MITT cohort, yielding estimated incidence rates of 3.4/100 person-years (95% CI: 2.8 to 4.0) and 3.3/100 person-years (95% CI: 2.8 to 3.9), respectively (**Figure 2A**). The primary efficacy outcome, estimated vaccine vs. placebo hazard ratio (HR), was 1.02 (95% CI: 0.81, 1.30) (**Table 2**). No differences in HIV incidence between vaccine and placebo groups were seen in secondary analyses over the full 36 months of follow-up (HR=1.05, 95% CI: 0.83 to 1.31), in the Month 6.5 at-risk cohort (HR =1.15, 95% CI: 0.84 to 1.58) (**Table 2**), or in additional secondary VE analyses, including PP VE (**Figures S2-S8**). No differential VE was evident over Months 0-24 by sex-at-birth (interaction P=0.92); estimated HR=1.03 among females (95% CI: 0.80 to 1.33) and 0.99 among males (95% CI: 0.50 to 1.98) (**Table 2** **and** **Figure 3A**). HIV incidence among females was 4.3/100 person-years (95% CI: 3.6 to 5.2) in the vaccine group and 4.2/100 person-years (95% CI: 3.5 to 5.0) in the placebo group, whereas among males the incidence was 1.3/100 person-years (95% CI: 0.7 to 2.0) amongst vaccine vs. 1.3/100 person-years (95% CI: 0.7 to 2.1) amongst placebo recipients (**Figure 3A**). Secondary analyses also included pre-specified assessment of potential vaccine effect modification by age, baseline HIV risk score, body mass index (BMI) and region over Months 0-24 among females. None of these factors were found to modify VE (multiplicity-adjusted interaction P values ≥0.09; **Table S12**).

**Table 2:** **Rate of HIV-1 infection and estimated hazard ratio for HIV-1 infection (Vaccine vs. Placebo)**. Data are shown for the Modified Intention-to-Treat (MITT) and the Month 6.5 At-Risk (M6.5AR) cohorts overall, by sex-at-birth and by follow-up period, and by age among females.

	Vaccine (n =2695)				Placebo (n = 2689)				Method^+^	Estimate
Cohort (time period)	No. evaluated	No. infect.	No. of person-yrs.	Rate no./pyrs. (%)	No. evaluated	No. infect.	No. of person-yrs.	Rate no./pyrs. (%)		HR (95% CI)
MITT cohort (Month 0-24)	2695	138	4098.3	0.034	2689	133	4052.7	0.033	Cox	1.02 (0.81, 1.30)
MITT cohort (Month 0-24)	2695	138	4098.3	0.034	2689	133	4052.7	0.033	CIR	1.03 (0.81, 1.31)
MITT cohort (Month 0-36)	2695	151	4477.9	0.034	2689	143	4438.7	0.032	Cox	1.05 (0.83, 1.31)
MITT cohort (Month 0-30*)	2695	147	4392.6	0.036	2689	141	4350.0	0.032	CIR	1.05 (0.81, 1.36)
M6.5AR cohort^ǂ^ (Month 6.5-24)	2430	83	2804.0	0.030	2393	71	2760.6	0.026	Cox	1.15 (0.84, 1.58)
M6.5AR cohort^ǂ^ (Month 6.5-24)	2430	83	2804.0	0.030	2393	71	2760.6	0.026	CIR	1.12 (0.81, 1.54)
Sex at birth, MITT cohort (Month 0-24)										
Female	1887	122	2819.9	0.043	1886	117	2787.3	0.042	Cox	1.03 (0.80, 1.33)
Male	808	16	1278.4	0.013	803	16	1265.5	0.013	Cox	0.99 (0.50, 1.98)
Age, Female MITT cohort (Month 0-24 )										
≤ 25 years	1264	87	1832.1	0.047	1267	80	1829.6	0.044	Cox	1.08 (0.80, 1.47)
> 25 years	623	35	987.8	0.035	619	37	957.7	0.039	Cox	0.92 (0.58, 1.46)

ǂ The Month 6.5 at-risk (M6.5AR) cohort is the set of modified intent-to-treat (MITT) participants on-study and HIV-1-negative at Month 6.5, at-risk of subsequent HIV-1 infection.

+ Hazard ratio (HR) (Vaccine vs. Placebo) is estimated using the Cox proportional hazards model or using the Cumulative Incidence Ratio (CIR).

* Cumulative Incidence Ratio is estimated over the time period where there are at least 150 participants of either sex-at-birth at risk.

#### Post-infection viral load

Among the 294 MITT participants diagnosed with HIV-1 infection over the full 36 months of follow-up (**Table S13**), mean log10 viral loads at the time of diagnosis were similar between vaccine and placebo recipients (4.82 log_10_ copies/mL, 95% CI: 4.61 to 5.02 and 4.64 log_10_ copies/mL, 95% CI: 4.45 to 4.84; **Figure S9**). The median time to antiretroviral therapy initiation was 13 weeks in the vaccine vs. 14 weeks in the placebo group (P = 0.5, log-rank test; **Figure S10**).

#### PrEP/PEP use

Despite PrEP being freely available, overall PrEP/PEP use was low: 120 (3.2%) females and 52 (3.2%) males self-reporting PrEP use and 91 (2.4%) females and 80 (4.9%) males self-reporting PEP use at any time (**Table S14**). Of the 2,405 DBS samples collected, TFV-DP levels were detectable in 51 (2.12%), reaching effective levels in five (**Tables S15, S16**). Overall, an estimated 2.89% person-years had detectable PrEP/PEP in the placebo and 1.99% in the vaccine group.

#### Safety, reactogenicity and death

Product administration errors were rare: 3 of 2704 participants randomized to vaccine and 2 of 2700 participants randomized to placebo received incorrect product. Participants receiving vaccine at any visit were analyzed as vaccinees for safety analyses. Vaccinations were safe and well-tolerated (**Tables S17-S19**). Vaccine recipients were more likely than placebo recipients to report reactogenicity (46.2% vs. 32.8%, P<0.001), with pain and/or tenderness most frequently reported for vaccine (23.0%) and headache reported most frequently by placebo recipients (15.7%). Most symptoms were mild. AEs were well-balanced between groups (**Table S20**) with AEs “related” to study product uncommon but more frequent in vaccine recipients (1.4% vs. 0.4% of placebo recipients, P<0.001). The few related AEs resulting in vaccination discontinuation included generalized rash (2 placebo, 1 vaccine), generalized urticaria (1 vaccine), cellulitis (1 vaccine), diarrhea (1 placebo), and headache (1 placebo).

#### Deaths

Eighteen deaths, all judged unrelated, were reported in 10 placebo and 8 vaccine recipients (Supplementary Materials).

#### Pregnancies

Only 163 pregnancies were reported (82 in vaccine, 81 in placebo recipients), yielding a 2.65% annual pregnancy rate. For 78 (48%) pregnancies, oral hormonal contraception was the method last reported. No congenital anomalies were reported.

## Discussion

We found no effect of the vaccine on HIV-1 acquisition in this well-powered study. The trial met the pre-specified non-efficacy stopping criteria in an interim analysis in January 2020 with no safety concerns. The DSMB recommended that vaccinations be stopped, participants unblinded and followed for one year post-last vaccination.

HVTN 702 differed from RV144 in inserts (ZM96 vs. 92TH023; 1086 and TV1 vs. A244, MN), adjuvants (MF59 vs. alum), and an additional boost (Month 18).^8-10^ Differing patterns of immunogenicity were seen in studies comparing the two regimens in SA. HVTN 100 evaluated immunogenicity of the HVTN 702 regimen and was compared to HVTN 097, a study that evaluated the RV144 regimen in SA.^11^ Binding and functional antibodies to gp120/gp140 antigens and T-cell responses to vaccine-matched peptide pools were of greater magnitude in HVTN 100, while the V2 region antibody responses, which were important correlates of risk in RV144,^12^ were higher for the RV144 regimen as assessed in both RV144 and HVTN 097.^13^ The substantial differences in antibody specificities induced by vaccination in these two regimens suggest that viral sequences or adjuvants may influence the elicitation of V2-specific antibodies, identified as a correlate of decreased HIV-1 risk.^14^

HIV-1 acquisition, and potentially HIV-1 exposure, was markedly higher in our study compared to RV144. Genital tract inflammation was likely prevalent, given the high STI prevalence among women.^15^ The 4.2% HIV-1 incidence in HVTN 702 females was 14 times higher than seen in RV144 females (0.30%). This incidence reflects hyperendemic HIV transmission in the community, likely from high frequency of acute infections and low rates of viral suppression.^16^ In RV144, lower VE was observed among participants at high risk of HIV-1 acquisition compared to those at low/moderate risk.^17^ In nonhuman primates vaccinated with ALVAC gp120, better protection from experimental mucosal infection was achieved with low-dose compared to high-dose challenge,^18^ providing a possible explanation about the role that “force” of infection plays in overcoming vaccine-induced immunity. We did not find efficacy in any subgroup, even those determined low risk for HIV-1 acquisition based on STI and behavioral data, however the eligibility criteria and burden of HIV in SA would suggest that few “low risk” women were enrolled.

The significantly greater genetic diversity of the sub-Saharan African subtype C epidemic compared to that of the AE epidemic in Thailand 15 years ago when RV144 was conducted is also likely to have played a role in the differential efficacy between the trials. VE in RV144 was found to depend on viral genetics, especially the match of exposing HIV-1 to vaccine insert at Env position 169 in the V2 loop.^19^ Studies suggest that the frequency of 169-match to the HVTN 702 vaccine is much less common in SA vs. Thailand,^20^ as is match of the V1V2 region of the HVTN 702 vaccine components with circulating SA sequences compared to their RV144 Thailand counterparts based on HIV sequence data from the Los Alamos National Labs (LANL) database^21^ (Supplementary Materials, **Figure S11**).

Host genetic factors that may influence VE also differ between the South African and Thai populations. RV144 data suggest that VE depends on *FCGR2C* and HLA-A*02 genotype.^22-24^ Recent data suggest SA has relatively low prevalence of FCR and HLA class I genotypes associated with high VE in RV144^25-27^ (Supplementary Materials, **Table S21**).

Limitations to our study include the inability to directly compare the RV144 and HVTN 702 regimens within the same study to address differences in the vector, adjuvants, and proteins. The absence of an available immunological biomarker to predict protection further compounds our ability to infer whether differences in efficacy are explained by observed differences in immunogenicity or by other factors such as infection force and viral diversity. Additional studies on the immunology and viral sequences are underway to improve our understanding of the results and implications for the field.

Given the many differences between the HVTN 702 and RV144 studies — between the vaccines and the immune responses they generated; the differences in the level of viral exposure; the extent of matching between the vaccines and the exposing viruses; and in host genetics and other host factors − isolating which factor or combination of factors is responsible for the different efficacy results will be challenging.

## Conclusion

Despite promising immunogenicity, this canarypox/protein prime/boost HIV vaccine regimen was not efficacious. The high HIV-1 incidence observed in this study illustrates an unrelenting epidemic, especially amongst young women. More than ever, a vaccine to prevent HIV-1 acquisition is needed.

## Disclosure

Disclosure forms provided by the authors are available with the full text of this article at NEJM.org.

## References

[1] UNAIDS. UNAIDS Fact Sheet 2018. Geneva, Switzerland 2018.

[2] Human Sciences Research Council. The Fifth South African National HIV Prevalence, Incidence, Behaviour and Communication Survey, 2017: HIV Impact Assessment Summary Report. Cape Town, South Africa 2018.

[3] Rerks-Ngarm S, Pitisuttithum P, Nitayaphan S, et al. Vaccination with ALVAC and AIDSVAX to prevent HIV-1 infection in Thailand. N Engl J Med 2009;361:2209-20. 19843557 10.1056/NEJMoa0908492

[4] Gray G, Doherty T, Mohapi L, et al. HIV research in South Africa: Advancing life. South African Medical Journal 2019;109:36-40. 32252866 10.7196/SAMJ.2019.v109i11b.14264

[5] Laher F, Moodie Z, Cohen KW, et al. Safety and immune responses after a 12-month booster in healthy HIV-uninfected adults in HVTN 100 in South Africa: A randomized double-blind placebo-controlled trial of ALVAC-HIV (vCP2438) and bivalent subtype C gp120/MF59 vaccines. PLoS Med 2020;17:e1003038. 32092060 10.1371/journal.pmed.1003038 PMC7039414

[6] Gilbert PB, Grove D, Gabriel E, et al. A Sequential Phase 2b Trial Design for Evaluating Vaccine Efficacy and Immune Correlates for Multiple HIV Vaccine Regimens. Stat Commun Infect Dis 2011;3. 10.2202/1948-4690.1037 PMC3502884 23181167

[7] Holm S. A Simple Sequentially Rejective Multiple Test Procedure. Scandinavian Journal of Statistics 1979;6:65-70.

[8] O'Hagan DT. MF59 is a safe and potent vaccine adjuvant that enhances protection against influenza virus infection. Expert Rev Vaccines 2007;6:699-710. 17931151 10.1586/14760584.6.5.699

[9] O'Hagan DT, Ott GS, De Gregorio E, Seubert A. The mechanism of action of MF59 - an innately attractive adjuvant formulation. Vaccine 2012;30:4341-8. 22682289 10.1016/j.vaccine.2011.09.061

[10] Shen X, Laher F, Moodie Z, et al. HIV-1 Vaccine Sequences Impact V1V2 Antibody Responses: A Comparison of Two Poxvirus Prime gp120 Boost Vaccine Regimens. Sci Rep 2020;10:2093. 32034163 10.1038/s41598-020-57491-z PMC7005751

[11] Bekker LG, Moodie Z, Grunenberg N, et al. Subtype C ALVAC-HIV and bivalent subtype C gp120/MF59 HIV-1 vaccine in low-risk, HIV-uninfected, South African adults: a phase 1/2 trial. Lancet HIV 2018;5:e366-e78. 29898870 10.1016/S2352-3018(18)30071-7 PMC6028742

[12] Gottardo R, Bailer RT, Korber BT, et al. Plasma IgG to linear epitopes in the V2 and V3 regions of HIV-1 gp120 correlate with a reduced risk of infection in the RV144 vaccine efficacy trial. PLoS One 2013;8:e75665. 24086607 10.1371/journal.pone.0075665 PMC3784573

[13] Gray GE, Huang Y, Grunenberg N, et al. Immune correlates of the Thai RV144 HIV vaccine regimen in South Africa. Sci Transl Med 2019;11. 10.1126/scitranslmed.aax1880 PMC7199879 31534016

[14] Haynes BF, Gilbert PB, McElrath MJ, et al. Immune-correlates analysis of an HIV-1 vaccine efficacy trial. N Engl J Med 2012;366:1275-86. 22475592 10.1056/NEJMoa1113425 PMC3371689

[15] Passmore JA, Jaspan HB, Masson L. Genital inflammation, immune activation and risk of sexual HIV acquisition. Curr Opin HIV AIDS 2016;11:156-62. 26628324 10.1097/COH.0000000000000232 PMC6194860

[16] Powers KA, Ghani AC, Miller WC, et al. The role of acute and early HIV infection in the spread of HIV and implications for transmission prevention strategies in Lilongwe, Malawi: a modelling study. Lancet 2011;378:256-68. 21684591 10.1016/S0140-6736(11)60842-8 PMC3274419

[17] Robb ML, Rerks-Ngarm S, Nitayaphan S, et al. Risk behaviour and time as covariates for efficacy of the HIV vaccine regimen ALVAC-HIV (vCP1521) and AIDSVAX B/E: a post-hoc analysis of the Thai phase 3 efficacy trial RV 144. Lancet Infect Dis 2012;12:531-7. 22652344 10.1016/S1473-3099(12)70088-9 PMC3530398

[18] Vaccari M, Keele BF, Bosinger SE, et al. Protection afforded by an HIV vaccine candidate in macaques depends on the dose of SIVmac251 at challenge exposure. J Virol 2013;87:3538-48. 23325681 10.1128/JVI.02863-12 PMC3592147

[19] Rolland M, Edlefsen PT, Larsen BB, et al. Increased HIV-1 vaccine efficacy against viruses with genetic signatures in Env V2. Nature 2012;490:417-20. 22960785 10.1038/nature11519 PMC3551291

[20] Rademeyer C, Korber B, Seaman MS, et al. Features of Recently Transmitted HIV-1 Clade C Viruses that Impact Antibody Recognition: Implications for Active and Passive Immunization. PLoS Pathog 2016;12:e1005742. 27434311 10.1371/journal.ppat.1005742 PMC4951126

[21] Los Alamos National Labs (Accessed September 28, 2020, at http://www.hiv.lanl.gov/.)

[22] Li SS, Gilbert PB, Tomaras GD, et al. FCGR2C polymorphisms associate with HIV-1 vaccine protection in RV144 trial. J Clin Invest 2014;124:3879-90. 25105367 10.1172/JCI75539 PMC4151214

[23] Gartland AJ, Li S, McNevin J, et al. Analysis of HLA A*02 association with vaccine efficacy in the RV144 HIV-1 vaccine trial. J Virol 2014;88:8242-55. 24829343 10.1128/JVI.01164-14 PMC4135964

[24] Prentice HA, Tomaras GD, Geraghty DE, et al. HLA class II genes modulate vaccineinduced antibody responses to affect HIV-1 acquisition. Sci Transl Med 2015;7:296ra112. 10.1126/scitranslmed.aab4005 PMC4911012 26180102

[25] Lassauniere R, Tiemessen CT. Variability at the FCGR locus: characterization in Black South Africans and evidence for ethnic variation in and out of Africa. Genes Immun 2016;17:93 104. 26673965 10.1038/gene.2015.60

[26] Hertz T, Logan MG, Rolland M, et al. A study of vaccine-induced immune pressure on breakthrough infections in the Phambili phase 2b HIV-1 vaccine efficacy trial. Vaccine 2016;34:5792-801. 27756485 10.1016/j.vaccine.2016.09.054 PMC5309337

[27] Tshabalala M, Mellet J, Pepper MS. Human Leukocyte Antigen Diversity: A Southern African Perspective. J Immunol Res 2015;2015:746151. 26347896 10.1155/2015/746151 PMC4549606

