# Supplementary material for: Vaccine Efficacy of ALVAC-HIV and Bivalent Subtype C gp120/MF59 in Adults
Source: N Engl J Med. Author manuscript; Available in PMC 2021 Mar 25. (PMC7888373; doi:10.1056/NEJMoa2031499)
Supplement: Supplementary file 1 [file NEJM-2021-2031499-s1.pdf]

Supplementary Materials for HVTN 702 Primary Manuscript

HVTN 702 Study Team

08 January, 2021

Contents

1 HVTN 702 Study Team **4**

2 Supplementary Methods and Results **4**

2.1 Safety Analysis Methods 4

2.2 Sample Size Calculations 4

2.3 Baseline Participant Characteristics 6

2.4 Baseline Risk Score 13

2.5 Study Loss to Follow-Up 14

2.6 Adherence to Protocol 16

2.7 Additional Details on Primary Analyses of Vaccine Efficacy 18

2.8 Secondary Analyses of Vaccine Efficacy 19

2.8.1 Vaccine Efficacy over Time 19

2.8.2 Per-Protocol Vaccine Efficacy 24

2.8.3 Variation in Vaccine Efficacy by Baseline Subject Characteristics 26

2.9 Analyses of Post-Infection Outcomes 28

2.10 Use of Pre-Exposure Prophylaxis (PrEP) 31

2.11 Additional Safety Data and Analyses 33

3 South African HIV-1 Sequence Data **39**

4 South African Host Genetics **39**

References **40**

List of Tables

S1 HVTN 702 study team investigators and their institutional affliations. 4

S2 Estimated power to detect different levels of VE(0-24) based on a sample size of n=2700/group 5

S3 Estimated power to detect different levels of VE(0-24) based on a sample size of n=2700/group, for various levels of annual HIV-1 incidence rates in the placebo group 5
S4 Distribution of all baseline variables that were eligible for the HIV risk model, by treatment group in participants female at birth in the MITT cohort. N (%) for each category. 6
S5 Distribution of all baseline variables that were eligible for the HIV risk model, by treatment group in participants male at birth in the MITT cohort. N (%) for each category. 9
S6 Baseline STI test results by treatment group and sex-at-birth, among all MITT participants enrolled after protocol version 2 went into effect (N=4622) 11
S7 Number (%) of participants enrolled by site and by sex-at-birth. Each site had an operational target for enrollment. 12
S8 Distribution of baseline safety measurements by treatment group among enrolled participants female at birth 13
S9 Distribution of baseline safety measurements by treatment group among enrolled participants male at birth 13
S10 Visit retention by treatment group among enrolled participants (N=5404) 16
S11 Treatment adherence by vaccination visit and treatment group among enrolled participants (N=5404) 18
S12 Results of secondary analyses evaluating modification of VE by pre-specified baseline covariates among participants female at birth. The number of participants and number of HIV-1 infection events are shown by covariate strata. 27
S13 Post-infection visit retention among MITT HIV-1 infected participants 28
S14 Number (%) of enrolled participants self-reporting PrEP or PEP use at any time during the study 31
S15 PrEP/PEP use among enrolled participants by treatment group. Time period for self-reported usage is at the time of specimen collection. 31
S16 PrEP/PEP use among enrolled participants by sex at birth. Time period for self-reported usage is at the time of specimen collection. 32
S17 Local reactogenicities, systemic reactogenicities, and adverse events experienced by enrolled participants. Cells show the number (percent) of participants who experienced an event of a given severity across all vaccinations. Each participant was counted only once under the maximum severity of the events experienced. 33
S18 Rates of aggregate safety endpoints by treatment received among enrolled participants, and the results of Barnard's test comparing event rates between treatment groups 34
S19 Listing of adverse events of special interest (AESIs)[1] by treatment received (N=5404) among all enrolled participants 35
S20 Grade 1-5 adverse events by system organ class, severity, and treatment received, ordered by decreasing frequency in safety cohort (N=5404) 36
S21 Low prevalence of 2 linked Fc gamma receptor SNPs in South African population 40

List of Figures

S1 Cumulative incidence of loss to follow-up by treatment group among enrolled participants . . 15
S2 Cumulative HIV-1 risk difference over time, overall in MITT cohort 19

S3 Cumulative HIV-1 risk difference over time, females at birth in MITT cohort 20
S4 Cumulative HIV-1 risk difference over time, males at birth in MITT cohort 21
S5 Estimated instantaneous hazard ratio (vaccine vs. placebo) over time, overall in MITT cohort 22
S6 Estimated instantaneous hazard ratio (vaccine vs. placebo) over time, females at birth in MITT cohort 23
S7 Estimated instantaneous hazard ratio (vaccine vs. placebo) over time, males at birth in MITT cohort 24
S8 Estimated HIV-1 relative risk (vaccine vs. placebo) for each 3-monthly time bin; the dashed lines show the linear interpolation between time bins. 25
S9 Distribution of pre-ART viral load by treatment group and post-infection visit among MITT infected participants. 29
S10 Kaplan-Meier plot for time to ART initiation among MITT infected participants. Log-rank test was used to compare event-time distributions between treatment groups. 30
S11 Proportion of amino acid sites in V1V2 where circulating HIV-1 sequences in South Africa vs. Thailand match the components of the HVTN 702 vs. RV144 HIV vaccines. Estimates are based on n = 213 sequences from South Africa, 2009-2017; and n = 145 sequences from Thailand, 2003-2006. Match frequency is shown for the ALVAC inserts, the vaccine proteins, and for the consensus sequences for South Africa and Thailand. 39

1 HVTN 702 Study Team

Table S1: HVTN 702 study team investigators and their institutional affliations.

Name	Affiliation
Sheetal Kassim, MBBCH	Cape Town - Emavundleni Clinical Research Site, SA
Amy Ward, MBBCH	Cape Town - Khayelitsha Clinical Research Site, SA
Graeme Meintjes, MBBCH, MPH, PhD	Cape Town - Khayelitsha Clinical Research Site, SA
Dishiki Kalonji, MBBCH	Durban - Isipingo Clinical Research Site, SA
Nishanta Singh, MBBCH	Durban - Verulam Clinical Research Site, SA
Nivashnee Naicker, MBBCH	Durban - eThekwini Clinical Research Site, SA
Craig Innes, MD	Klerksdorp Clinical Research Site, SA
Philippus Kotze, MBBCH, MD	Ladysmith Clinical Research Site, SA
Maphoshane Nchabeleng, MBChB, Mmed	Medunsa Clinical Research Site, SA
Pamela Mda, MBBCH	Mthatha Clinical Research Site, SA
Thozama Dubula, MBBCH	Mthatha Clinical Research Site, SA
William Brumskine, MD	Rustenburg Clinical Research Site, SA
Mookho Mahlaleha, MBChB, MPH	Soshanguve Clinical Research Site, SA
Fatima Laher, MBBCH	Soweto - Bara Clinical Research Site, SA
Erica Lazarus, MBBCH	Soweto - Kliptown Clinical Research Site, SA
Modulakgotla Sebe, MBBCH	Tembisa Clinical Research Site, SA

2 Supplementary Methods and Results

2.1 Safety Analysis Methods

Participants were observed for 30 minutes after vaccinations and recorded solicited local and systemic symptoms (reactogenicity) for 3 days on a diary card after each vaccination with adverse events (AEs) recorded until 30 days after each vaccination.

Safety analyses included all enrolled participants, all of whom received at least one injection as this was concurrent with enrollment, and were performed according to the treatment received ("as treated"). Rates of reactogenicity and adverse events were compared between treatment groups using Barnard's exact test.

2.2 Sample Size Calculations

For the comparisons of HIV-1 infection rate between the vaccine and placebo groups over Months 0-24, we assumed a 4% annual HIV-1 incidence in the placebo group, a 20-month enrollment period with a uniform enrollment rate that is halved in the first 3 months, halved VE in the first 6 months, and a 5% annual loss to follow-up incidence. Under these assumptions and the sequential monitoring for potential harm, non-efficacy, and high efficacy, n = 2700 per group was selected to ensure approximately 90% power to detect vaccine efficacy from enrollment through 24 months [VE(0-24)] of at least 50% using a 1-sided alpha = 0.025-level log-rank test (versus the null hypothesis of H0: VE(0-24) $\leq$ 25%). Table S2 provides estimates of power to detect varying levels of VE(0-24), under these same assumptions.

To assess the impact of potential emerging prevention modalities on the study power, Table S3 reports the estimated power to detect VE(0-24) of 50% (versus the null hypothesis of H0: VE(0-24) $\leq$ 25%) for annual HIV-1 incidence rates in the placebo group ranging from 2.0% to 4.5%, holding the other assumptions and sequential monitoring the same as above. The range of annual HIV-1 incidence rates might reflect various levels of PrEP use. For example, assuming a placebo-group annual incidence of 4% without PrEP, a placebo-group annual incidence of 3% could arise from one extreme where 25% of the person-years of follow-up in

Table S2: Estimated power to detect different levels of VE(0-24) based on a sample size of n=2700/group

True Average VE(0-24)	Power to reject null: VE(0-24) <= 25%
30%	7
40%	45
50%	90
60%	100
70%	100
80%	100

the trial are under PrEP use and PrEP has 100% efficacy, the other extreme where 100% of the person-years of follow-up are under PrEP use and PrEP has 25% efficacy, or something in between such as 31% of the person-years of follow-up are under PrEP use and PrEP has 80% efficacy. Table S3 indicates that the study had power above 80% to detect VE(0-24) of 50% or higher (against the null of VE(0-24) $\leq$ 25%) for incidence as low as 3% annually.

Table S3: Estimated power to detect different levels of VE(0-24) based on a sample size of n=2700/group, for various levels of annual HIV-1 incidence rates in the placebo group

Incidence Rate	Power to reject null: VE(0-24) <= 25% when true average VE(0-24) = 50%
2.0%	65%
2.5%	75%
3.0%	81%
3.5%	86%
4.0%	91%
4.5%	93%

To enhance the likelihood that the background incidence assumption is valid and thereby to preserve study power to assess vaccine efficacy, while still maintaining representation of both sexes, no more than 35% and no fewer than 30% males (ie, persons assigned male-sex-at-birth) were enrolled.

2.3 Baseline Participant Characteristics

Table S4: Distribution of all baseline variables that were eligible for the HIV risk model, by treatment group in participants female at birth in the MITT cohort. N (%) for each category.

	Total (N=3773)	Vaccine (N=1887)	Placebo (N=1886)
Age at enrollment			
<=21	1114 (29.53%)	543 (28.78%)	571 (30.28%)
22-25	1417 (37.56%)	721 (38.21%)	696 (36.90%)
>25	1242 (32.92%)	623 (33.02%)	619 (32.82%)
Median (Min, Max)	24 (18, 35)	24 (18, 35)	23 (18, 35)
Body Mass Index categorization			
<18.5	133 (3.53%)	56 (2.97%)	77 (4.08%)
18.5-<25	1401 (37.13%)	724 (38.37%)	677 (35.90%)
25-<30	974 (25.82%)	490 (25.97%)	484 (25.66%)
>=30	1265 (33.53%)	617 (32.70%)	648 (34.36%)
Heterosexual orientation			
Yes	3684 (97.64%)	1840 (97.51%)	1844 (97.77%)
No	89 (2.36%)	47 (2.49%)	42 (2.23%)
Region categorization of enrollment site‡			
Central	1804 (47.81%)	906 (48.01%)	898 (47.61%)
KZN	1233 (32.68%)	614 (32.54%)	619 (32.82%)
Western/Eastern Cape	736 (19.51%)	367 (19.45%)	369 (19.57%)
Married or have main sex partner†			
Yes	3341 (88.55%)	1679 (88.98%)	1662 (88.12%)
No	240 (6.36%)	122 (6.47%)	118 (6.26%)
Missing	192 (5.09%)	86 (4.56%)	106 (5.62%)
Living with main sex partner†			
Yes	530 (14.05%)	239 (12.67%)	291 (15.43%)
No	2811 (74.50%)	1440 (76.31%)	1371 (72.69%)
Not Applicable	240 (6.36%)	122 (6.47%)	118 (6.26%)
Missing	192 (5.09%)	86 (4.56%)	106 (5.62%)
Main sex partner has other partners†			
Yes/Don't Know	2680 (71.03%)	1360 (72.07%)	1320 (69.99%)
No	661 (17.52%)	319 (16.91%)	342 (18.13%)
Not Applicable	240 (6.36%)	122 (6.47%)	118 (6.26%)
Missing	192 (5.09%)	86 (4.56%)	106 (5.62%)
First sexual intercourse before 16 years of age†			
Yes	506 (13.41%)	252 (13.35%)	254 (13.47%)
No	3077 (81.55%)	1551 (82.19%)	1526 (80.91%)
Mising	190 (5.04%)	84 (4.45%)	106 (5.62%)
Oldest sex partner older than 26*			
Yes	2586 (68.54%)	1301 (68.95%)	1285 (68.13%)
No	1182 (31.33%)	584 (30.95%)	598 (31.71%)
Missing	5 (0.13%)	2 (0.11%)	3 (0.16%)
Num. sex acts in past month >= 7*			
Yes	1907 (50.54%)	949 (50.29%)	958 (50.80%)
No	1864 (49.40%)	938 (49.71%)	926 (49.10%)
Missing	2 (0.05%)	0 (0.00%)	2 (0.11%)
Num. sex partners in past month >= 2*			
Yes	1888 (50.04%)	939 (49.76%)	949 (50.32%)
No	1885 (49.96%)	948 (50.24%)	937 (49.68%)
Babies alive at birth >= 2†			

Table S4: Distribution of all baseline variables that were eligible for the HIV risk model, by treatment group in participants female at birth in the MITT cohort. N (%) for each category. *(continued)*

	Total (N=3773)	Vaccine (N=1887)	Placebo (N=1886)
Yes	944 (25.02%)	472 (25.01%)	472 (25.03%)
No	2642 (70.02%)	1331 (70.54%)	1311 (69.51%)
Mising	187 (4.96%)	84 (4.45%)	103 (5.46%)
Anal sex*			
Yes	166 (4.40%)	76 (4.03%)	90 (4.77%)
No	3599 (95.39%)	1808 (95.81%)	1791 (94.96%)
Mising	8 (0.21%)	3 (0.16%)	5 (0.27%)
Condom use always			
Yes	209 (5.54%)	88 (4.66%)	121 (6.42%)
No	3563 (94.43%)	1798 (95.28%)	1765 (93.58%)
Mising	1 (0.03%)	1 (0.05%)	0 (0.00%)
Vaginal drying agent inserted for sex*			
Yes	96 (2.54%)	54 (2.86%)	42 (2.23%)
No	3670 (97.27%)	1830 (96.98%)	1840 (97.56%)
Missing	7 (0.19%)	3 (0.16%)	4 (0.21%)
Unprotected sex with alcohol use*			
Yes	1694 (44.90%)	835 (44.25%)	859 (45.55%)
No	2076 (55.02%)	1051 (55.70%)	1025 (54.35%)
Missing	3 (0.08%)	1 (0.05%)	2 (0.11%)
Sex with HIV+ partner*			
Yes	1713 (45.40%)	861 (45.63%)	852 (45.17%)
No	2054 (54.44%)	1021 (54.11%)	1033 (54.77%)
Missing	6 (0.16%)	5 (0.26%)	1 (0.05%)
Unprotected sex with HIV+ partner*			
Yes/Don't Know	28 (0.74%)	17 (0.90%)	11 (0.58%)
No	2073 (54.94%)	1035 (54.85%)	1038 (55.04%)
Not Asked	1671 (44.29%)	834 (44.20%)	837 (44.38%)
Missing	1 (0.03%)	1 (0.05%)	0 (0.00%)
Any STI			
Yes	1003 (26.58%)	514 (27.24%)	489 (25.93%)
No	2372 (62.87%)	1182 (62.64%)	1190 (63.10%)
Missing	398 (10.55%)	191 (10.12%)	207 (10.98%)
Exchange services for sex*			
Yes	789 (20.91%)	384 (20.35%)	405 (21.47%)
No	2973 (78.80%)	1497 (79.33%)	1476 (78.26%)
Missing	11 (0.29%)	6 (0.32%)	5 (0.27%)
Genital discharge*			
Yes	258 (6.84%)	129 (6.84%)	129 (6.84%)
No	3505 (92.90%)	1755 (93.00%)	1750 (92.79%)
Missing	10 (0.27%)	3 (0.16%)	7 (0.37%)
Genital sores*			
Yes	98 (2.60%)	51 (2.70%)	47 (2.49%)
No	3670 (97.27%)	1833 (97.14%)	1837 (97.40%)
Missing	5 (0.13%)	3 (0.16%)	2 (0.11%)
Urban living area†			
Yes	3033 (80.39%)	1520 (80.55%)	1513 (80.22%)
No	553 (14.66%)	283 (15.00%)	270 (14.32%)
Mising	187 (4.96%)	84 (4.45%)	103 (5.46%)
Formal dwelling†			

Table S4: Distribution of all baseline variables that were eligible for the HIV risk model, by treatment group in participants female at birth in the MITT cohort. N (%) for each category. *(continued)*

	Total (N=3773)	Vaccine (N=1887)	Placebo (N=1886)
Yes	2886 (76.49%)	1463 (77.53%)	1423 (75.45%)
No	700 (18.55%)	340 (18.02%)	360 (19.09%)
Mising	187 (4.96%)	84 (4.45%)	103 (5.46%)
Dwelling has 3 or more utilities†			
Yes	3105 (82.30%)	1545 (81.88%)	1560 (82.71%)
No	481 (12.75%)	258 (13.67%)	223 (11.82%)
Mising	187 (4.96%)	84 (4.45%)	103 (5.46%)
Birth control method at baseline§			
Implant w/o Injectable	426 (11.29%)	212 (11.23%)	214 (11.35%)
Injectable	3042 (80.63%)	1521 (80.60%)	1521 (80.65%)
Oral w/o (Injectable/Implant)	191 (5.06%)	95 (5.03%)	96 (5.09%)
Other	114 (3.02%)	59 (3.13%)	55 (2.92%)

* Timeframe for question is the previous 30 days.

† Question introduced after study began and asked retrospectively when required. It is missing for 172 females who were lost to follow-up prior to its introduction.

‡ Central sites include Klerksdorp, Medunsa, Rustenburg, Soshanguve, Soweto-Bara, Soweto-Kliptown, and Tembisa. KZN sites include Durban-eThekwini, Durban-Isipingo, Durban-Verulam, and Ladysmith. Western/Eastern Cape sites include Cape Town-Emavundleni, Cape Town-Khayelitsha, and Mthatha

§ More than one contraceptive method could be specified, so percentages may add up to more than 100.

Table S5: Distribution of all baseline variables that were eligible for the HIV risk model, by treatment group in participants male at birth in the MITT cohort. N (%) for each category.

	Total (N=1611)	Vaccine (N=808)	Placebo (N=803)
Age at enrollment			
<26	755 (46.87%)	383 (47.40%)	372 (46.33%)
>=26	856 (53.13%)	425 (52.60%)	431 (53.67%)
Median (Min, Max)	26 (18, 35)	26 (18, 35)	26 (18, 35)
Body Mass Index categorization			
<18.5	232 (14.40%)	114 (14.11%)	118 (14.69%)
18.5-<25	1121 (69.58%)	558 (69.06%)	563 (70.11%)
25-<30	193 (11.98%)	96 (11.88%)	97 (12.08%)
>=30	65 (4.03%)	40 (4.95%)	25 (3.11%)
Heterosexual orientation			
Yes	1419 (88.08%)	703 (87.00%)	716 (89.17%)
No	192 (11.92%)	105 (13.00%)	87 (10.83%)
Region categorization of enrollment site‡			
Central	831 (51.58%)	415 (51.36%)	416 (51.81%)
KZN	522 (32.40%)	261 (32.30%)	261 (32.50%)
Western/Eastern Cape	258 (16.01%)	132 (16.34%)	126 (15.69%)
Married or have main sex partner†			
Yes	1375 (85.35%)	684 (84.65%)	691 (86.05%)
No	180 (11.17%)	97 (12.00%)	83 (10.34%)
Missing	56 (3.48%)	27 (3.34%)	29 (3.61%)
Living with main sex partner†			
Yes	278 (17.26%)	144 (17.82%)	134 (16.69%)
No	1096 (68.03%)	540 (66.83%)	556 (69.24%)
Not Applicable	180 (11.17%)	97 (12.00%)	83 (10.34%)
Missing	57 (3.54%)	27 (3.34%)	30 (3.74%)
Main sex partner has other partners†			
Yes/Don't Know	939 (58.29%)	460 (56.93%)	479 (59.65%)
No	434 (26.94%)	223 (27.60%)	211 (26.28%)
Not Applicable	180 (11.17%)	97 (12.00%)	83 (10.34%)
Missing	58 (3.60%)	28 (3.47%)	30 (3.74%)
Num. sex acts in past month >= 8*			
Yes	835 (51.83%)	428 (52.97%)	407 (50.68%)
No	776 (48.17%)	380 (47.03%)	396 (49.32%)
Num. sex partners in past month >= 2*			
Yes	1115 (69.21%)	561 (69.43%)	554 (68.99%)
No	496 (30.79%)	247 (30.57%)	249 (31.01%)
Sex with HIV+ partner*			
Yes	868 (53.88%)	427 (52.85%)	441 (54.92%)
No	740 (45.93%)	379 (46.91%)	361 (44.96%)
Missing	3 (0.19%)	2 (0.25%)	1 (0.12%)
Unprotected sex with HIV+ partner*			
Yes/Don't Know	22 (1.37%)	13 (1.61%)	9 (1.12%)
No	761 (47.24%)	393 (48.64%)	368 (45.83%)
Not Asked	824 (51.15%)	401 (49.63%)	423 (52.68%)
Missing	4 (0.25%)	1 (0.12%)	3 (0.37%)
Unprotected sex with alcohol use*			
Yes	958 (59.47%)	479 (59.28%)	479 (59.65%)
No	648 (40.22%)	326 (40.35%)	322 (40.10%)
Mising	5 (0.31%)	3 (0.37%)	2 (0.25%)

Table S5: Distribution of all baseline variables that were eligible for the HIV risk model, by treatment group in participants male at birth in the MITT cohort. N (%) for each category. *(continued)*

	Total (N=1611)	Vaccine (N=808)	Placebo (N=803)
Anal sex*			
Yes	249 (15.46%)	136 (16.83%)	113 (14.07%)
No	1354 (84.05%)	668 (82.67%)	686 (85.43%)
Mising	8 (0.50%)	4 (0.50%)	4 (0.50%)
Condom use always			
Yes	139 (8.63%)	78 (9.65%)	61 (7.60%)
No	1471 (91.31%)	729 (90.22%)	742 (92.40%)
Mising	1 (0.06%)	1 (0.12%)	0 (0.00%)
Fully circumcised¶			
Yes	858 (53.26%)	429 (53.09%)	429 (53.42%)
No	548 (34.02%)	279 (34.53%)	269 (33.50%)
Missing	205 (12.73%)	100 (12.38%)	105 (13.08%)
Exchange services for sex*			
Yes	255 (15.83%)	128 (15.84%)	127 (15.82%)
No	1350 (83.80%)	678 (83.91%)	672 (83.69%)
Missing	6 (0.37%)	2 (0.25%)	4 (0.50%)
Any STI			
Yes	228 (14.15%)	118 (14.60%)	110 (13.70%)
No	1016 (63.07%)	511 (63.24%)	505 (62.89%)
Missing	367 (22.78%)	179 (22.15%)	188 (23.41%)
Genital discharge*			
Yes	22 (1.37%)	12 (1.49%)	10 (1.25%)
No	1585 (98.39%)	794 (98.27%)	791 (98.51%)
Missing	4 (0.25%)	2 (0.25%)	2 (0.25%)
Genital sores*			
Yes	26 (1.61%)	18 (2.23%)	8 (1.00%)
No	1583 (98.26%)	790 (97.77%)	793 (98.75%)
Missing	2 (0.12%)	0 (0.00%)	2 (0.25%)
Urban living area+			
Yes	1339 (83.12%)	677 (83.79%)	662 (82.44%)
No	219 (13.59%)	106 (13.12%)	113 (14.07%)
Mising	53 (3.29%)	25 (3.09%)	28 (3.49%)
Formal dwelling+			
Yes	1297 (80.51%)	653 (80.82%)	644 (80.20%)
No	261 (16.20%)	130 (16.09%)	131 (16.31%)
Mising	53 (3.29%)	25 (3.09%)	28 (3.49%)
Dwelling has 3 or more utilities+			
Yes	1394 (86.53%)	703 (87.00%)	691 (86.05%)
No	164 (10.18%)	80 (9.90%)	84 (10.46%)
Mising	53 (3.29%)	25 (3.09%)	28 (3.49%)

* Timeframe for question is the previous 30 days.

† Question introduced after study began and asked retrospectively when required. It is missing for 52 males who were lost to follow-up prior to its introduction.

‡ Central sites include Klerksdorp, Medunsa, Rustenburg, Soshanguve, Soweto-Bara, Soweto-Kliptown, and Tembisa. KZN sites include Durban-eThekwini, Durban-Isipingo, Durban-Verulam, and Ladysmith. Western/Eastern Cape sites include Cape Town-Emavundleni, Cape Town-Khayelitsha, and Mthatha

§ More than one contraceptive method could be specified, so percentages may add up to more than 100.

¶ Circumcision status at enrollment was assessed by physical exam.

Table S6: Baseline STI test results by treatment group and sex-at-birth, among all MITT participants enrolled after protocol version 2 went into effect (N=4622)

	Female at Birth			Male at Birth		
	Total	Placebo	Vaccine	Total	Placebo	Vaccine
Any STI[1]						
Positive	1003 (29.7%)	489 (29.1%)	514 (30.3%)	228 (18.3%)	110 (17.9%)	118 (18.7%)
Negative	2372 (70.2%)	1190 (70.8%)	1182 (69.7%)	1016 (81.6%)	505 (82.1%)	511 (81.1%)
Not done/Indeterminate	2 (0.1%)	2 (0.1%)	0 (0.0%)	1 (0.1%)	0 (0.0%)	1 (0.2%)
Syphilis[2]						
Positive	44 (1.3%)	23 (1.4%)	21 (1.2%)	24 (1.9%)	14 (2.3%)	10 (1.6%)
Negative	3310 (98.0%)	1648 (98.0%)	1662 (98.0%)	1211 (97.3%)	596 (96.9%)	615 (97.6%)
Not done/Indeterminate	23 (0.7%)	10 (0.6%)	13 (0.8%)	10 (0.8%)	5 (0.8%)	5 (0.8%)
Trichomonas[3]						
Positive	189 (5.6%)	94 (5.6%)	95 (5.6%)	-	-	-
Negative	3101 (91.8%)	1545 (91.9%)	1556 (91.7%)	-	-	-
Not done/Indeterminate	87 (2.6%)	42 (2.5%)	45 (2.7%)	-	-	-
N.Gonorrhea[4]						
Positive	178 (5.3%)	89 (5.3%)	89 (5.2%)	38 (3.1%)	19 (3.1%)	19 (3.0%)
Negative	3124 (92.5%)	1556 (92.6%)	1568 (92.5%)	1183 (95.0%)	583 (94.8%)	600 (95.2%)
Not done/Indeterminate	75 (2.2%)	36 (2.1%)	39 (2.3%)	24 (1.9%)	13 (2.1%)	11 (1.7%)
C.Trachomatis[4]						
Positive	773 (22.9%)	368 (21.9%)	405 (23.9%)	199 (16.0%)	96 (15.6%)	103 (16.3%)
Negative	2534 (75.0%)	1281 (76.2%)	1253 (73.9%)	1017 (81.7%)	502 (81.6%)	515 (81.7%)
Not done/Indeterminate	70 (2.1%)	32 (1.9%)	38 (2.2%)	29 (2.3%)	17 (2.8%)	12 (1.9%)

[1] Indicates positivity to any of the listed STIs for which a participant was tested.

[2] Both non-treponemal and treponemal test must be positive for a positive diagnosis. Test performed on blood samples.

[3] Only done for females at birth. Test performed on cervical/vaginal swab.

[4] Test performed on cervical/vaginal swab, urine or rectal swab.

Table S7: Number (%) of participants enrolled by site and by sex-at-birth. Each site had an operational target for enrollment.

Site	Sex-at-birth	N	%
Cape Town-Emavundleni	Female	380	7.00
	Male	125	2.30
	Total	505	9.34
Cape Town-Khayelitsha	Female	297	5.50
	Male	132	2.40
	Total	429	7.94
Durban-Isipingo	Female	345	6.40
	Male	147	2.70
	Total	492	9.10
Durban-Verulam	Female	356	6.60
	Male	155	2.90
	Total	511	9.46
Durban-eThekwini	Female	259	4.80
	Male	95	1.80
	Total	354	6.55
Klerksdorp	Female	237	4.40
	Male	116	2.10
	Total	353	6.53
Ladysmith	Female	279	5.20
	Male	128	2.40
	Total	407	7.53
Medunsa	Female	215	4.00
	Male	118	2.20
	Total	333	6.16
Mthatha	Female	61	1.10
	Male	3	0.10
	Total	64	1.18
Rustenburg	Female	231	4.30
	Male	127	2.40
	Total	358	6.62
Soshanguve	Female	341	6.30
	Male	142	2.60
	Total	483	8.94
Soweto-Bara	Female	380	7.00
	Male	105	1.90
	Total	485	8.97
Soweto-Kliptown	Female	222	4.10
	Male	118	2.20
	Total	340	6.29
Tembisa	Female	183	3.40
	Male	107	2.00
	Total	290	5.37
All Sites	Female	3786	70.06
	Male	1618	29.94
	Total	5404	100.00

Table S8: Distribution of baseline safety measurements by treatment group among enrolled participants female at birth

Baseline Measurement	All (N=3786)	Placebo (N=1893)	Vaccine (N=1893)
Median hemoglobin (g/dL), IQR	13.3 (12.5, 14.0)	13.3 (12.5, 14.0)	13.3 (12.5, 14.0)
Median WBC (10^3/mm^3), IQR	6.4 (5.3, 7.8)	6.3 (5.3, 7.8)	6.5 (5.3, 7.9)

Note: IQR = interquartile range WBC = white blood cell count

Table S9: Distribution of baseline safety measurements by treatment group among enrolled participants male at birth

Baseline Measurement	All (N=1618)	Placebo (N=807)	Vaccine (N=811)
Median hemoglobin (g/dL), IQR	15.2 (14.4, 16.0)	15.2 (14.4, 16.0)	15.2 (14.4, 16.0)
Median WBC (10^3/mm^3), IQR	5.4 (4.4, 6.7)	5.5 (4.4, 6.8)	5.3 (4.4, 6.6)

Note: IQR = interquartile range WBC = white blood cell count

2.4 Baseline Risk Score

Risk scores were developed for males and females at birth, restricting to the MITT cohort and Month 0-24 follow-up. The variables listed in Tables S4 and S5 were eligible for the female and male risk scores respectively, as well as all two-way interactions of the above variables and all two-way interactions of each variable with treatment assignment. The function `missForest` from the `R` package of the same name was used for single imputation of missing values. Screening of variables for risk modeling was done separately for females and males. Univariate Cox proportional hazards models were fit using each of the individual variables and all of the interaction terms. Interactions between each variable and treatment assignment were screened by comparing models with both main effects and the two-way interaction to models with only the two main effects. All variables with an unadjusted likelihood ratio test p-value < 0.10 passed this screening. Any individual variables or interactions passing this screening, or any individual variables involved in interactions passing this screening, were 'screened-in' to the risk modeling. All pairwise interactions between this set of variables were added and were also eligible for the risk modeling. The function `cv.glmnet()` in the `R` package `glmnet` was used with lasso penalty to select the Cox proportional hazards regression model for predicting HIV-1 infection. Ten-fold cross-validation (CV) was used to select the optimal female model, with deviance loss function; five-fold CV was employed for the male risk model. The final baseline HIV risk score was defined as the linear predictor of the fitted Cox model. For females, the binary risk score categories correspond (as closely as possible) to values above vs. below the median, and for the 3-category score, the categories correspond (as closely as possible) to tertiles of the score. For males, the risk score takes only 4 unique values, and the binary score classifies 89% (11%) of males as low (high) risk.

For females, the variables selected into the risk score in the form of main effects or interactions were: prevalent STI, main sex partner has other partners (yes or don't know), geographic region (Central South Africa or KwaZulu-Natal vs. Western/Eastern Cape), unprotected sex with alcohol use, and unprotected sex with an HIV-positive partner. The risk score was modestly associated with HIV-1 risk among females, with an estimated hazard ratio per standard-deviation of 1.55 (95% CI: 1.36 to 1.78). The cross-validated estimated C-index for the risk score was 0.60.

For males, the variables selected into the risk score in the form of main effects or interactions were: anal sex, exchange of services for sex, and sexual orientation (heterosexual vs. non-heterosexual). The risk score was strongly associated with HIV-1 risk among males, with an estimated hazard ratio for high vs. low risk of 2.54 (95% CI: 2.03 to 3.17). The cross-validated estimated C-index for the risk score was 0.73.

2.5 Study Loss to Follow-Up

Figure S1 shows the cumulative rate of loss to follow-up by treatment group. A total of 292 (5%) of MITT participants terminated the study early. Participants who terminated early are considered to have been lost to follow-up immediately following the last completed study visit. Participants who missed all scheduled or interim visits in the past 35 weeks (8 months) and who missed their past 3 or more consecutive visits not including post-vaccination immunogenicity visits are also considered to have been lost to follow-up at the date of their last contact. Participants completing 24 months of follow-up HIV-1 negative at the Month 24 visit, or diagnosed with HIV-1 infection are censored at 24 months, or the time of HIV-1 diagnosis, respectively. The rate of loss-to-follow-up was similar in the two treatment groups.

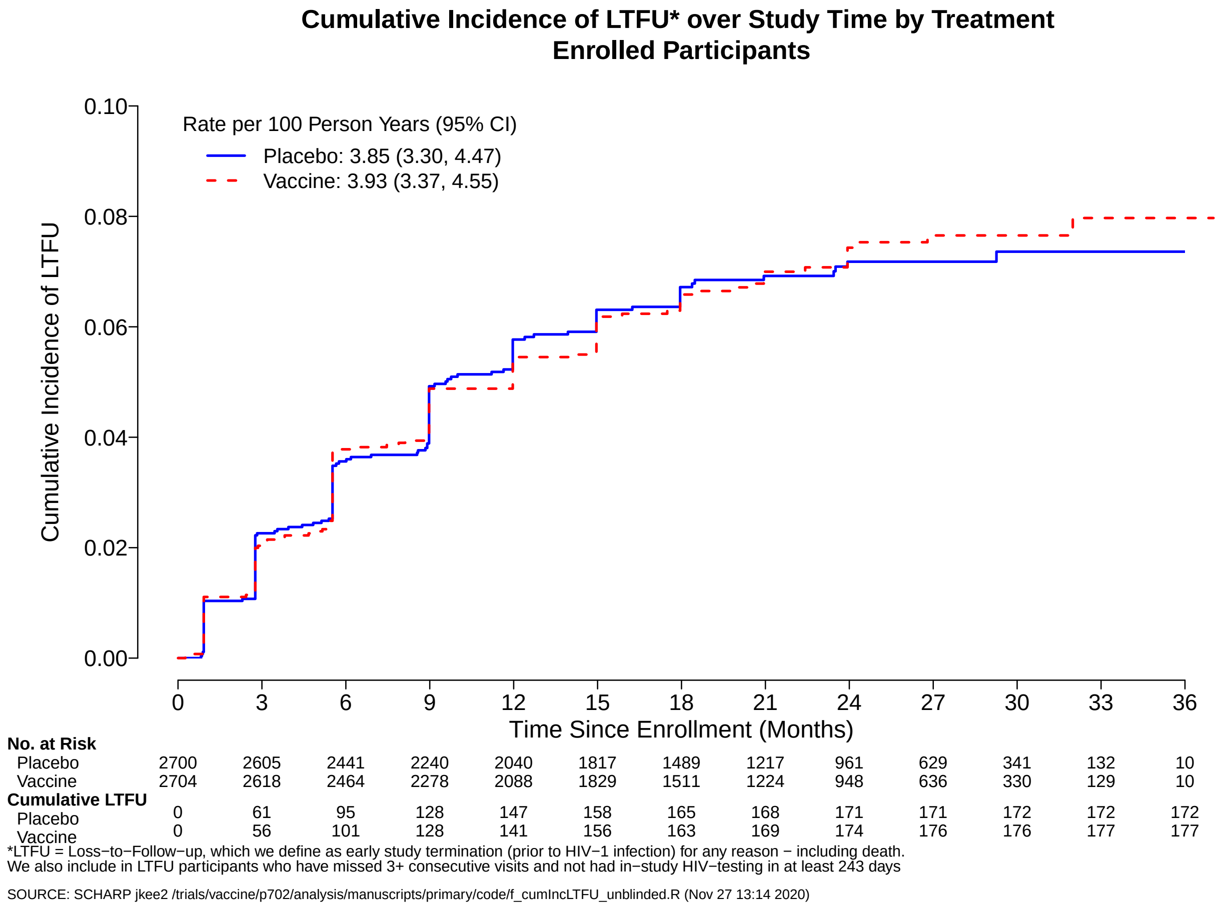

Figure S1: Cumulative incidence of loss to follow-up by treatment group among enrolled participants

2.6 Adherence to Protocol

Table S10: Visit retention by treatment group among enrolled participants (N=5404)

	Total	Placebo	Vaccine
Total Enrolled	5404	2700	2704
Month 1 / Vaccination 2			
Expected* for Visit	5388	2692	2696
Completed	5156 (95.7%)	2572 (95.5%)	2584 (95.8%)
Missed	230 (4.3%)	120 (4.5%)	110 (4.1%)
Terminated study prior to visit	2 (0.0%)	0 (0.0%)	2 (0.1%)
Month 3 / Vaccination 3			
Expected* for Visit	5387	2692	2695
Completed	5097 (94.6%)	2541 (94.4%)	2556 (94.8%)
Missed	279 (5.2%)	144 (5.3%)	135 (5.0%)
Terminated study prior to visit	11 (0.2%)	7 (0.3%)	4 (0.1%)
Month 6 / Vaccination 4			
Expected* for Visit	5339	2664	2675
Completed	4963 (93.0%)	2484 (93.2%)	2479 (92.7%)
Missed	324 (6.1%)	154 (5.8%)	170 (6.4%)
Terminated study prior to visit	52 (1.0%)	26 (1.0%)	26 (1.0%)
Month 6.5 / Post-Vaccination 4			
Expected* for Visit	5309	2655	2654
Completed	4741 (89.3%)	2385 (89.8%)	2356 (88.8%)
Missed	498 (9.4%)	233 (8.8%)	265 (10.0%)
Terminated study prior to visit	70 (1.3%)	37 (1.4%)	33 (1.2%)
Month 9			
Expected* for Visit	4954	2463	2491
Completed	4529 (91.4%)	2247 (91.2%)	2282 (91.6%)
Missed	319 (6.4%)	161 (6.5%)	158 (6.3%)
Terminated study prior to visit	106 (2.1%)	55 (2.2%)	51 (2.0%)
Month 12 / Vaccination 5			
Expected* for Visit	4539	2256	2283
Completed	4097 (90.3%)	2043 (90.6%)	2054 (90.0%)
Missed	296 (6.5%)	135 (6.0%)	161 (7.1%)
Terminated study prior to visit	146 (3.2%)	78 (3.5%)	68 (3.0%)
Month 12.5 / Post-Vaccination 5			
Expected* for Visit	4576	2269	2307
Completed	3985 (87.1%)	1976 (87.1%)	2009 (87.1%)
Missed	431 (9.4%)	207 (9.1%)	224 (9.7%)
Terminated study prior to visit	160 (3.5%)	86 (3.8%)	74 (3.2%)
Month 15			
Expected* for Visit	4185	2081	2104
Completed	3761 (89.9%)	1872 (90.0%)	1889 (89.8%)
Missed	247 (5.9%)	113 (5.4%)	134 (6.4%)
Terminated study prior to visit	177 (4.2%)	96 (4.6%)	81 (3.8%)
Month 18 / Vaccination 6			
Expected* for Visit	3606	1796	1810
Completed	3223 (89.4%)	1607 (89.5%)	1616 (89.3%)
Missed	198 (5.5%)	89 (5.0%)	109 (6.0%)
Terminated study prior to visit	185 (5.1%)	100 (5.6%)	85 (4.7%)
Month 18.5 / Post-Vaccination 6			
Expected* for Visit	3639	1815	1824

Table S10: Visit retention by treatment group among enrolled participants (N=5404) *(continued)*

	Total	Placebo	Vaccine
Completed	3120 (85.7%)	1557 (85.8%)	1563 (85.7%)
Missed	329 (9.0%)	157 (8.7%)	172 (9.4%)
Terminated study prior to visit	190 (5.2%)	101 (5.6%)	89 (4.9%)
Month 21			
Expected* for Visit	2949	1475	1474
Completed	2618 (88.8%)	1300 (88.1%)	1318 (89.4%)
Missed	167 (5.7%)	81 (5.5%)	86 (5.8%)
Terminated study prior to visit	164 (5.6%)	94 (6.4%)	70 (4.7%)
Month 24			
Expected* for Visit	2334	1170	1164
Completed	2052 (87.9%)	1020 (87.2%)	1032 (88.7%)
Missed	137 (5.9%)	68 (5.8%)	69 (5.9%)
Terminated study prior to visit	145 (6.2%)	82 (7.0%)	63 (5.4%)
Month 27			
Expected* for Visit	1786	900	886
Completed	1581 (88.5%)	792 (88.0%)	789 (89.1%)
Missed	90 (5.0%)	43 (4.8%)	47 (5.3%)
Terminated study prior to visit	115 (6.4%)	65 (7.2%)	50 (5.6%)
Month 30			
Expected* for Visit	1035	531	504
Completed	926 (89.5%)	473 (89.1%)	453 (89.9%)
Missed	51 (4.9%)	25 (4.7%)	26 (5.2%)
Terminated study prior to visit	58 (5.6%)	33 (6.2%)	25 (5.0%)
Month 33			
Expected* for Visit	498	255	243
Completed	443 (89.0%)	220 (86.3%)	223 (91.8%)
Missed	28 (5.6%)	18 (7.1%)	10 (4.1%)
Terminated study prior to visit	27 (5.4%)	17 (6.7%)	10 (4.1%)
Month 36			
Expected* for Visit	76	41	35
Completed	69 (90.8%)	36 (87.8%)	33 (94.3%)
Missed	2 (2.6%)	1 (2.4%)	1 (2.9%)
Terminated study prior to visit	5 (6.6%)	4 (9.8%)	1 (2.9%)

*Participants are considered expected for a visit when they reach the end of their visit window. HIV-1-infected participants are not expected for these visits, post-infection, but participants that withdraw from the study continue to be expected.

Table S11: Treatment adherence by vaccination visit and treatment group among enrolled participants (N=5404)

	All Expected Excluding Terminated		
	Total	Placebo	Vaccine
Month 0/Vaccination 1			
Expected*	5404	2700	2704
Received treatment	5404 (100.0%)	2700 (100.0%)	2704 (100.0%)
Missed visit and treatment	0 (0.0%)	0 (0.0%)	0 (0.0%)
Completed visit, Missed treatment	0 (0.0%)	0 (0.0%)	0 (0.0%)
Discontinued treatment**, on-study	0 (0.0%)	0 (0.0%)	0 (0.0%)
Month 1/Vaccination 2			
Expected*	5386	2692	2694
Received treatment	5105 (94.8%)	2549 (94.7%)	2556 (94.9%)
Missed visit and treatment	0 (0.0%)	0 (0.0%)	0 (0.0%)
Completed visit, Missed treatment	0 (0.0%)	0 (0.0%)	0 (0.0%)
Discontinued treatment**, on-study	0 (0.0%)	0 (0.0%)	0 (0.0%)
Month 3/Vaccination 3			
Expected*	5376	2685	2691
Received treatment	5007 (93.1%)	2495 (92.9%)	2512 (93.3%)
Missed visit and treatment	0 (0.0%)	0 (0.0%)	0 (0.0%)
Completed visit, Missed treatment	0 (0.0%)	0 (0.0%)	0 (0.0%)
Discontinued treatment**, on-study	6 (0.1%)	3 (0.1%)	3 (0.1%)
Month 6/Vaccination 4			
Expected*	5287	2638	2649
Received treatment	4842 (91.6%)	2422 (91.8%)	2420 (91.4%)
Missed visit and treatment	0 (0.0%)	0 (0.0%)	0 (0.0%)
Completed visit, Missed treatment	0 (0.0%)	0 (0.0%)	0 (0.0%)
Discontinued treatment**, on-study	11 (0.2%)	5 (0.2%)	6 (0.2%)
Month 12/Vaccination 5			
Expected*	4393	2178	2215
Received treatment	3971 (90.4%)	1978 (90.8%)	1993 (90.0%)
Missed visit and treatment	0 (0.0%)	0 (0.0%)	0 (0.0%)
Completed visit, Missed treatment	0 (0.0%)	0 (0.0%)	0 (0.0%)
Discontinued treatment**, on-study	18 (0.4%)	11 (0.5%)	7 (0.3%)
Month 18/Vaccination 6			
Expected*	3421	1696	1725
Received treatment	3092 (90.4%)	1537 (90.6%)	1555 (90.1%)
Missed visit and treatment	0 (0.0%)	0 (0.0%)	0 (0.0%)
Completed visit, Missed treatment	0 (0.0%)	0 (0.0%)	0 (0.0%)
Discontinued treatment**, on-study	29 (0.8%)	16 (0.9%)	13 (0.8%)

*HIV-1 infected participants are not considered expected post-infection. Participants who terminated early are also excluded from this table.
**Discontinued treatment prior to the indicated visit.

2.7 Additional Details on Primary Analyses of Vaccine Efficacy

As stated in the Methods section, the primary efficacy parameter [VE(0-24)] was estimated by using a Cox proportional hazards regression (stratified by sex-at-birth) and by a ratio of estimated cumulative incidences. Cumulative incidence was estimated separately by sex-at-birth and then the sex-specific estimates were combined using the stratified Aalen-Johansen estimator with a single failure type (Aalen, 1978); a Wald test was used for inference. While the cumulative incidence-based estimator of VE(0-24) was considered primary,

these estimates were nearly identical to the Cox regression-based estimates (see Table 2 in manuscript), and therefore the Cox estimates are reported throughout for consistency with the reporting of secondary VE analyses based on Cox regression.

2.8 Secondary Analyses of Vaccine Efficacy

2.8.1 Vaccine Efficacy over Time

To assess potential time-effects of VE, the transformed Nelson-Aalen cumulative hazard function was used to estimate cumulative HIV-1 incidence over time for the vaccine and placebo groups, and the additive difference in cumulative vaccine efficacy over time. The method of Parzen, Wei, and Ying (1997) was applied to obtain point-wise and simultaneous 95% confidence intervals. Figures S2-S4 show the estimated additive difference-based VE estimates over 0-24 months, for the MITT population at large and for females and males at birth in the MITT cohort.

To further explore potential time-variation in VE, instantaneous VE defined as one minus the instantaneous hazard ratio (vaccine/placebo) over time was estimated using the nonparametric kernel estimation method, with the asymptotic pointwise and simultaneous 95% confidence intervals calculated using the method of Gilbert et al. (2002). Figures S3-S5 show the estimated instantaneous VE over 0-24 months, for the MITT population at large and for females and males at birth in the MITT cohort.

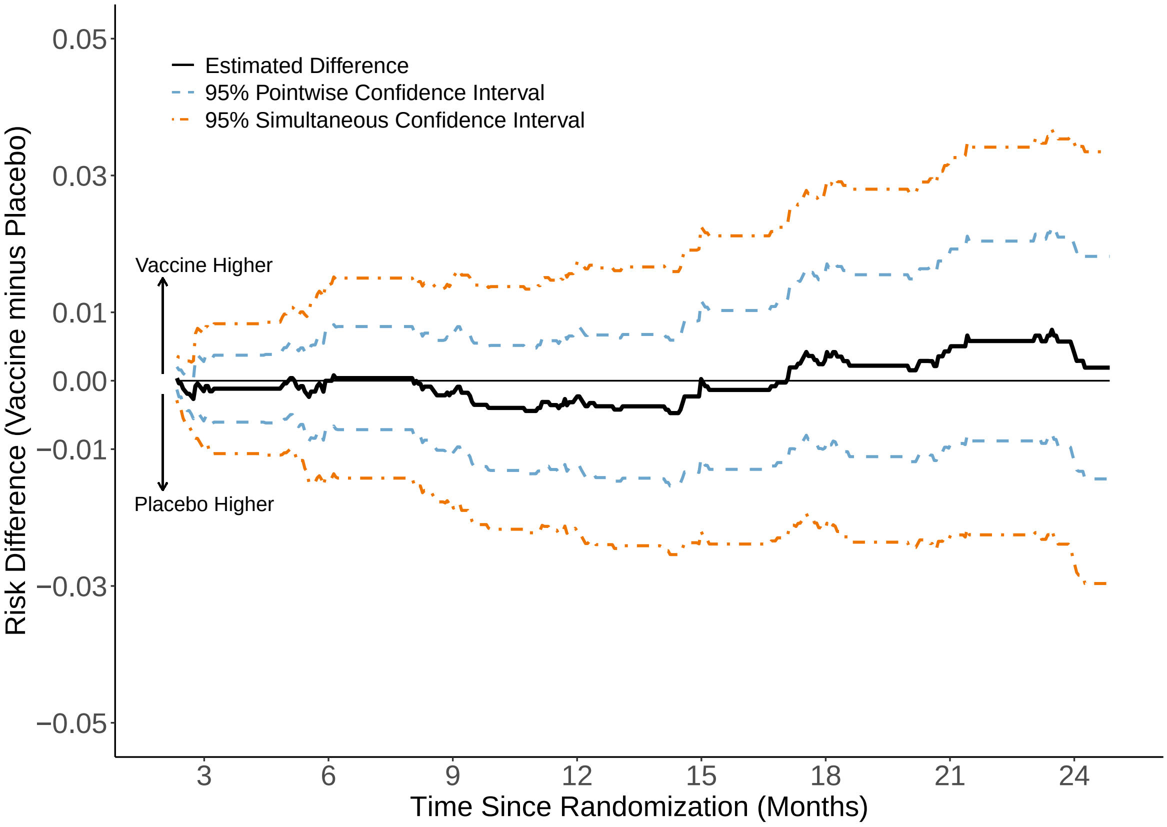

SOURCE: SCHARP jkee2 /trials/vaccine/p702/analysis/manuscripts/primary/code/f_risk_diff_VE_24.R (Sep 10 08:42 2020)

Figure S2: Cumulative HIV-1 risk difference over time, overall in MITT cohort

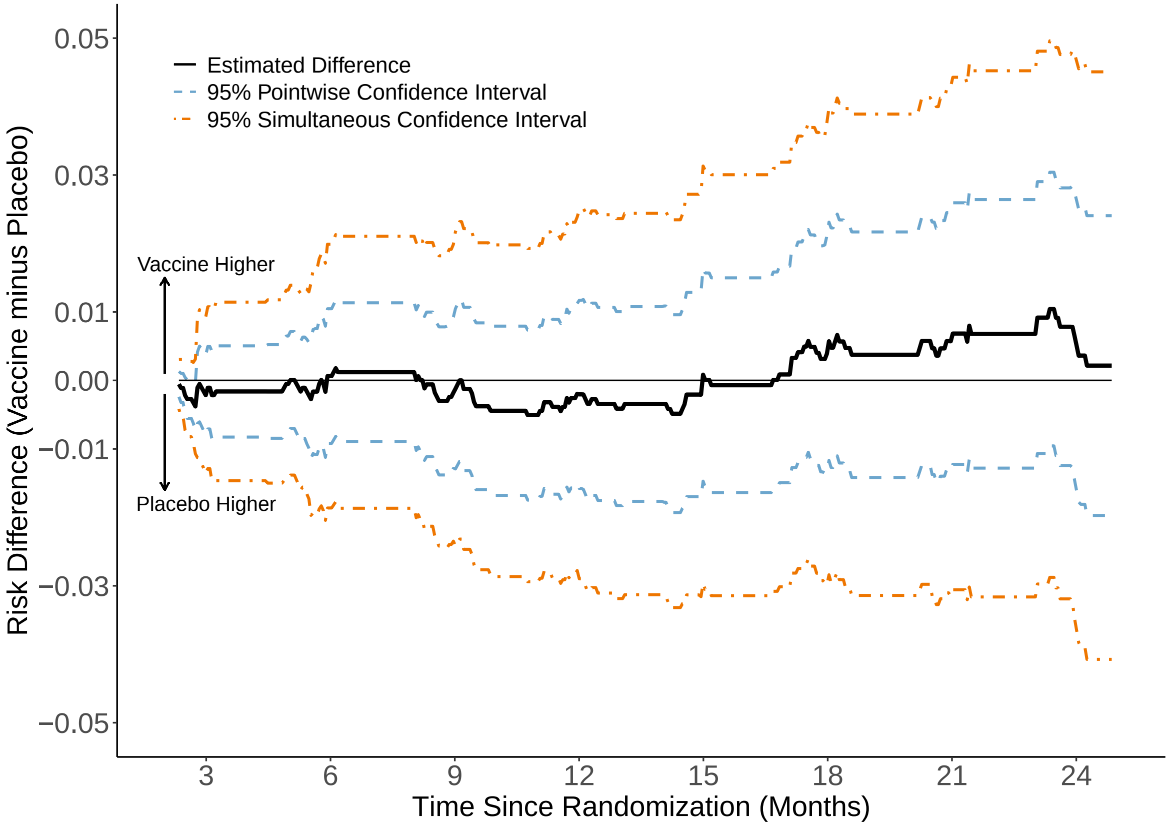

SOURCE: SCHARP jkee2 /trials/vaccine/p702/analysis/manuscripts/primary/code/f_risk_diff_VE_24_fem.R (Sep 10 08:42 2020)

Figure S3: Cumulative HIV-1 risk difference over time, females at birth in MITT cohort

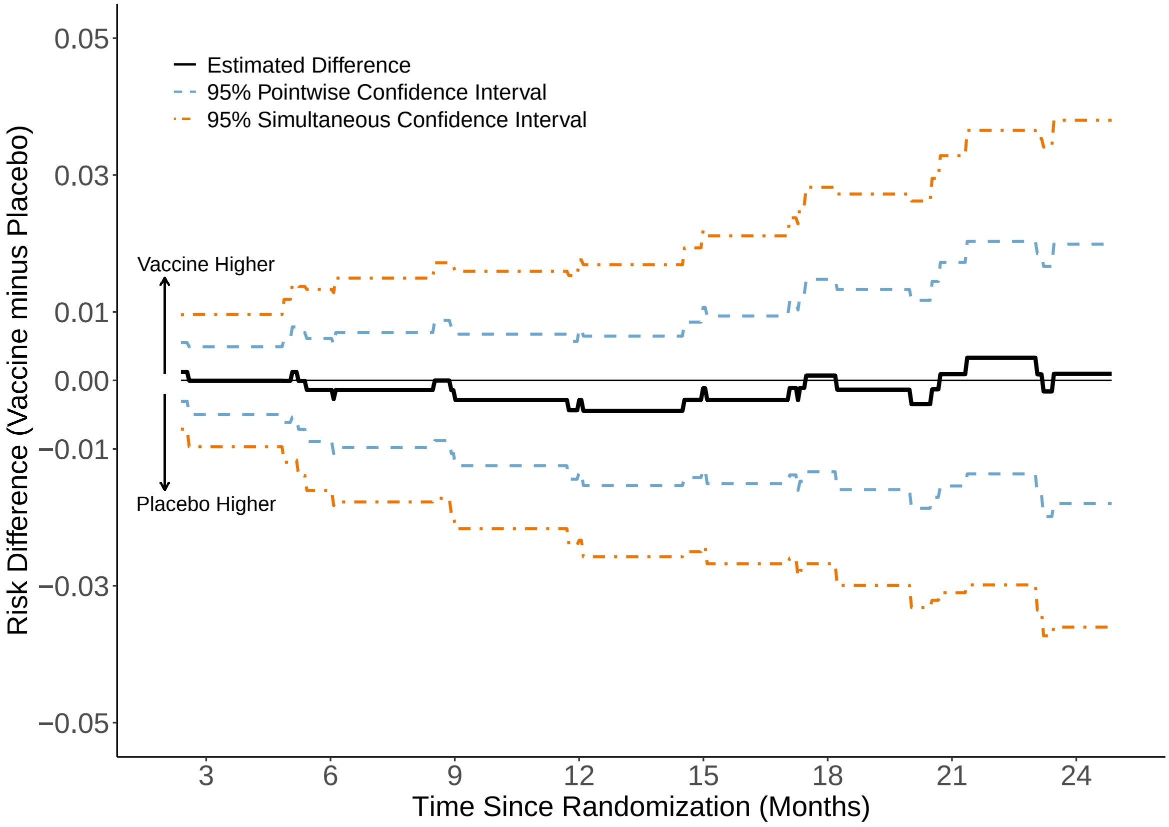

SOURCE: SCHARP jkee2 /trials/vaccine/p702/analysis/manuscripts/primary/code/f_risk_diff_VE_24_male.R (Sep 10 08:44 2020)

Figure S4: Cumulative HIV-1 risk difference over time, males at birth in MITT cohort

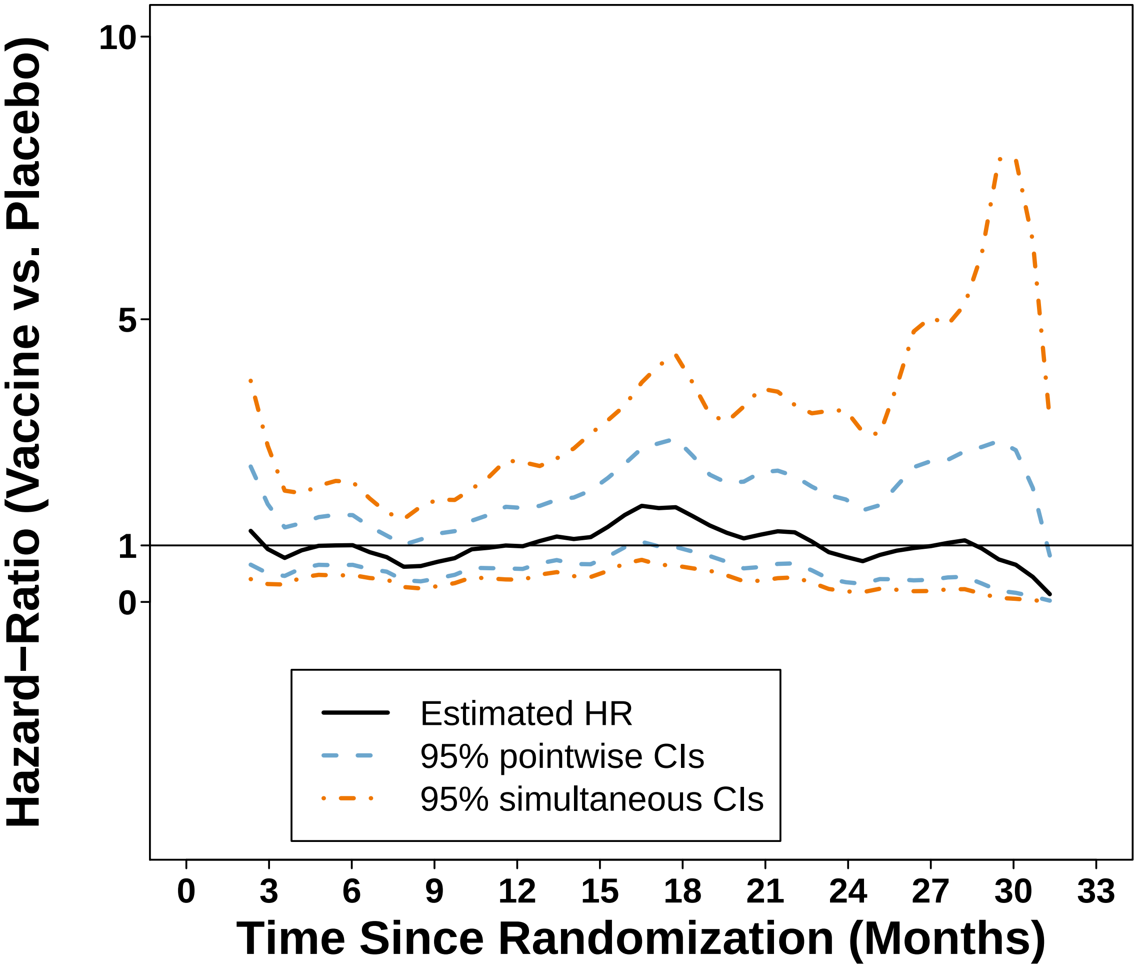

Figure S5: Estimated instantaneous hazard ratio (vaccine vs. placebo) over time, overall in MITT cohort

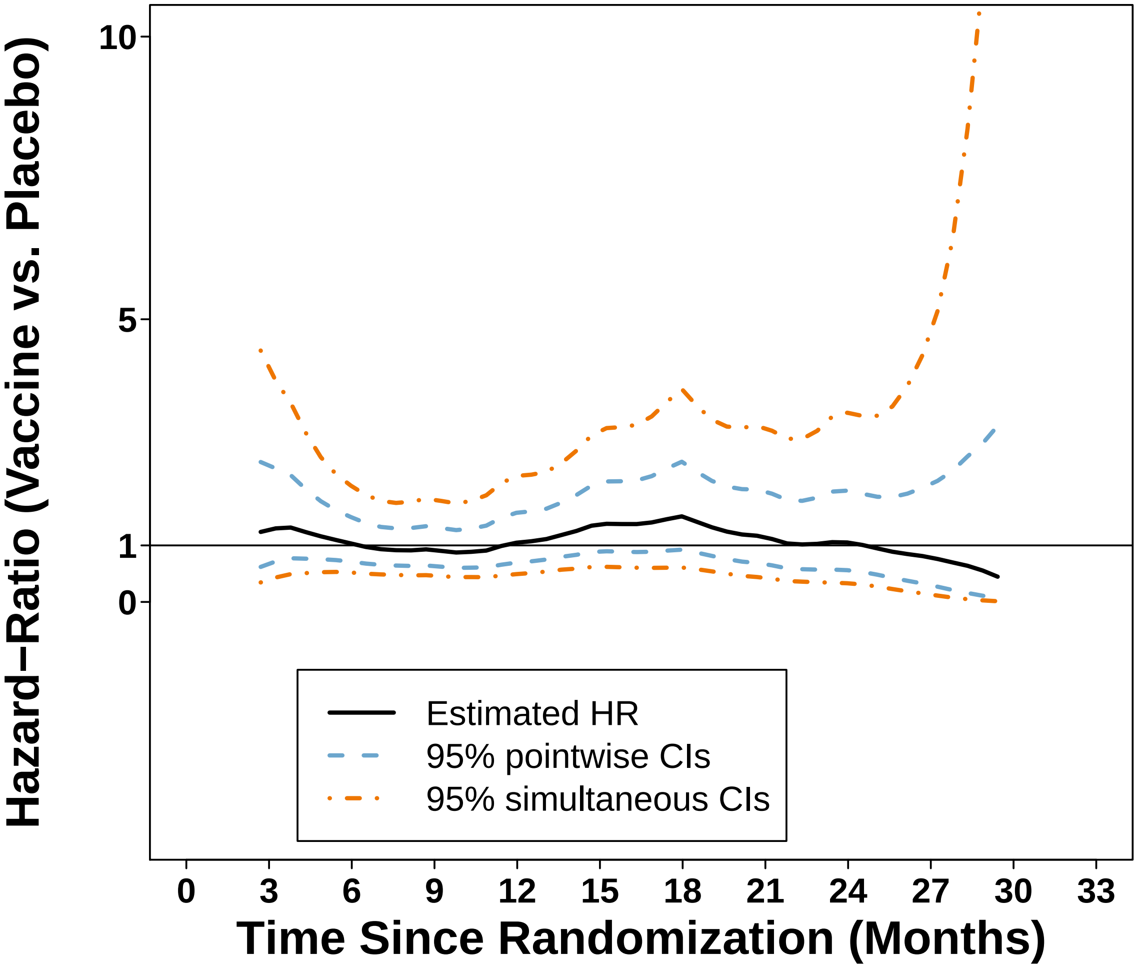

Figure S6: Estimated instantaneous hazard ratio (vaccine vs. placebo) over time, females at birth in MITT cohort

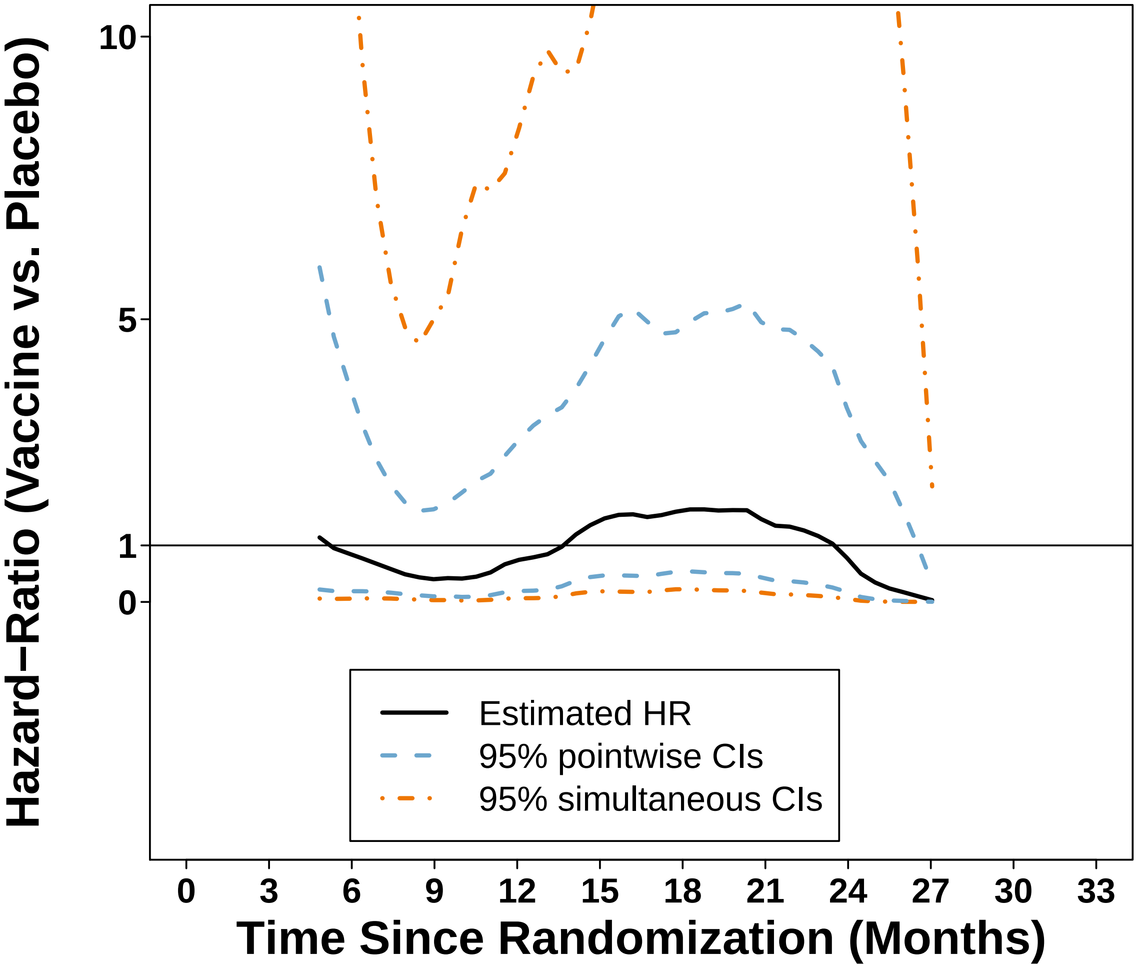

Figure S7: Estimated instantaneous hazard ratio (vaccine vs. placebo) over time, males at birth in MITT cohort

2.8.2 Per-Protocol Vaccine Efficacy

Per-protocol vaccine efficacy, measured by cumulative VE in the per-protocol population, was estimated using targeted minimum loss based estimation (TMLE) combined with super learning to adjust for participant's demographic and risk behavior information (Benkeser and Hejazi 2017; Benkeser, Carone, and Gilbert 2017; Benkeser, Gilbert, and Carone 2019). The estimated ratio of cumulative HIV-1 risk at 24 months (vaccine vs. placebo) was 0.90 (95% CI: 0.70, 1.17). Figure S8 shows the estimated relative risk for 3-monthly bins of time from Month 6.5 to Month 24.

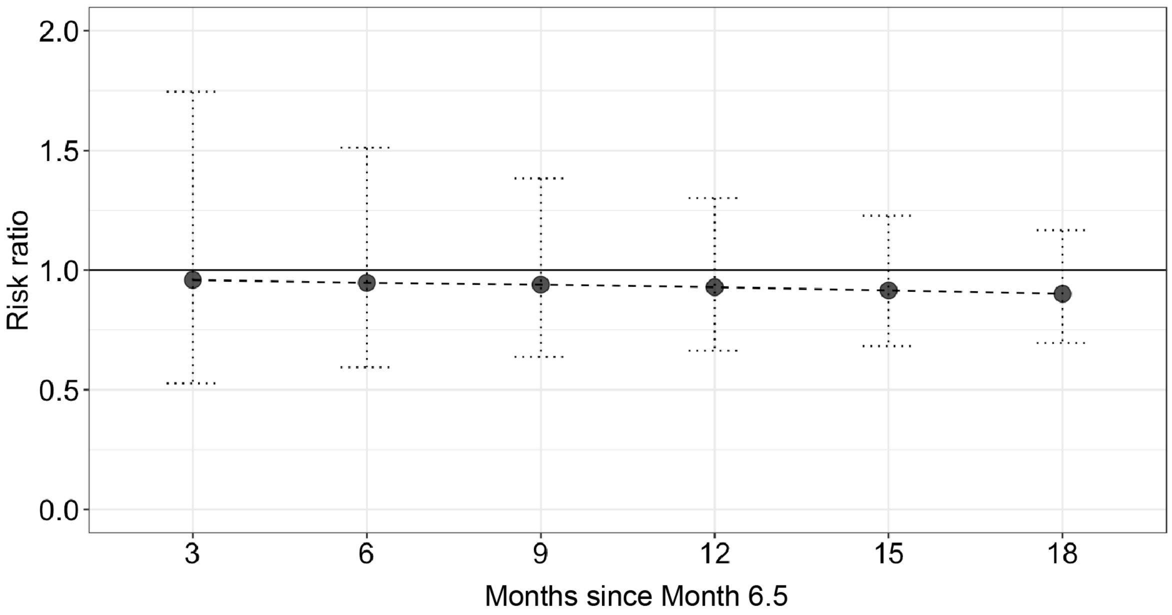

Figure S8: Estimated HIV-1 relative risk (vaccine vs. placebo) for each 3-monthly time bin; the dashed lines show the linear interpolation between time bins.

2.8.3 Variation in Vaccine Efficacy by Baseline Subject Characteristics

Secondary analyses were conducted to evaluate potential modification of VE among women by baseline participant characteristics. The relatively small number of men at birth enrolled, paired with the relatively low incidence in this population, precluded performing these analyses among men. For each baseline covariate, Wald tests for the treatment-by-covariate interaction were performed in the context of a Cox proportional hazards model that allows for different baseline hazards for the different strata of each categorical covariate. The interaction test p-values were adjusted for multiple comparisons using the Holm method (1979), controlling the family-wise error across all continuous and categorical covariates considered. Table S15 shows the number of HIV-1 infection events by covariate strata and the results of the interaction tests.

Table S12: Results of secondary analyses evaluating modification of VE by pre-specified baseline covariates among participants female at birth. The number of participants and number of HIV-1 infection events are shown by covariate strata.

	Num. Evaluated	Num. Infections (Placebo, Vaccine)	Nominal Interaction P-value*	Adjusted Interaction P-value†
Age (<= 25; > 25)	2531; 1242	(80, 87); (37, 35)	0.55	1.00
Age (<= 21; 22-25; > 25)	1114; 1417; 1242	(28, 48); (52, 39); (37, 35)	0.01	0.09
Age (continuous)	3773	(117, 122)	0.30	1.00
Region (KZN; other)	1233; 2540	(52, 58); (65, 64)	0.54	1.00
Region (KZN; Central; Western/Eastern Cape)	1233; 1804; 736	(52, 58); (48, 39); (17, 25)	0.18	1.00
BMI (< 25; >= 25)	1534; 2239	(55, 52); (62, 70)	0.33	1.00
BMI (<18.5; 18.5-<25; 25-<30; >=30)	133; 1401; 974; 1265	(8, 5); (47, 47); (19, 38); (43, 32)	0.08	0.74
Risk score (<= median; > median)	2251; 1522	(56, 50); (61, 72)	0.30	1.00
Risk score (low; med; high)	1067; 1184; 1522	(19, 21); (37, 29); (61, 72)	0.40	1.00
Risk score (continuous)	3773	(117, 122)	0.69	1.00

* Corresponds to testing null hypothesis of HR = 1 for interaction term

† Adjusted for multiplicity using Holm method, considering all 10 pre-specified analyses of interactions

2.9 Analyses of Post-Infection Outcomes

Participants who seroconverted over the course of the study were rolled into a post-infection follow-up schedule, with clinical and laboratory assessments at the time of HIV-1 diagnosis, and at Weeks 12 and 24 post-infection. Time of antiretroviral therapy (ART) initiation was captured using a case report form and is defined as the date of reported initiation of treatment.

Table S13: Post-infection visit retention among MITT HIV-1 infected participants

Post-Infection Visit	Total: Number completed visit / Number expected*	Placebo: Number completed visit / Number expected*	Vaccine: Number completed visit / Number expected*
Total Infected (N)	294	143	151
Week 12	195 / 257 (76.00%)	93 / 123 (76.00%)	102 / 134 (76.00%)
Week 24	161 / 213 (76.00%)	83 / 106 (78.00%)	78 / 107 (73.00%)

* Participants are expected when they reach the end of the visit window. Participants who withdraw continue to be expected and count against retention.

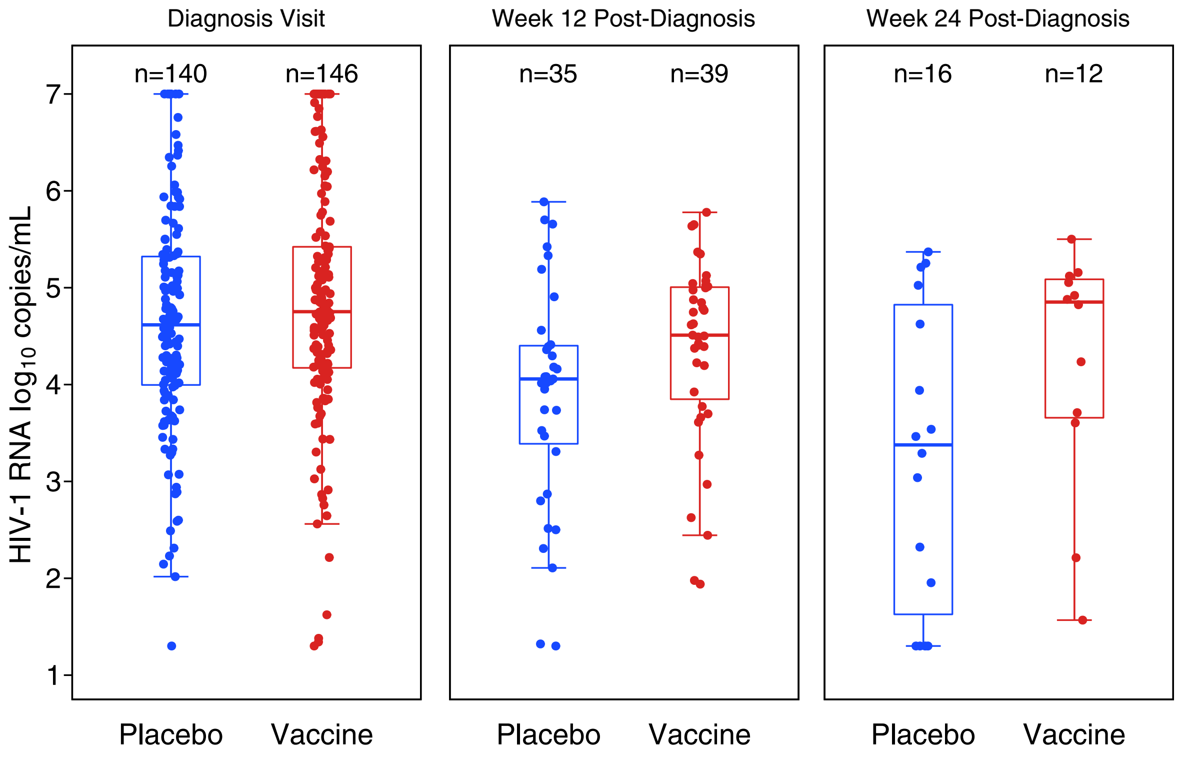

SOURCE: SCHARP jkee2 /trials/vaccine/p702/analysis/manuscripts/primary/code/f_preARTVL_bytrt.R (Jan 06 09:35 2021)

Figure S9: Distribution of pre-ART viral load by treatment group and post-infection visit among MITT infected participants.

Viral load measurements were censored (set to missing) after ART initiation. Mean viral loads at the HIV-1 diagnosis were similar between vaccine and placebo recipients (4.82 $\log_{10}$ copies/mL, 95% CI: 4.61 to 5.02 and 4.64 $\log_{10}$ copies/mL, 95% CI: 4.45 to 4.84. Testing was not done at other time points due to the small sample size attributable to ART initiation and loss-to-follow-up.

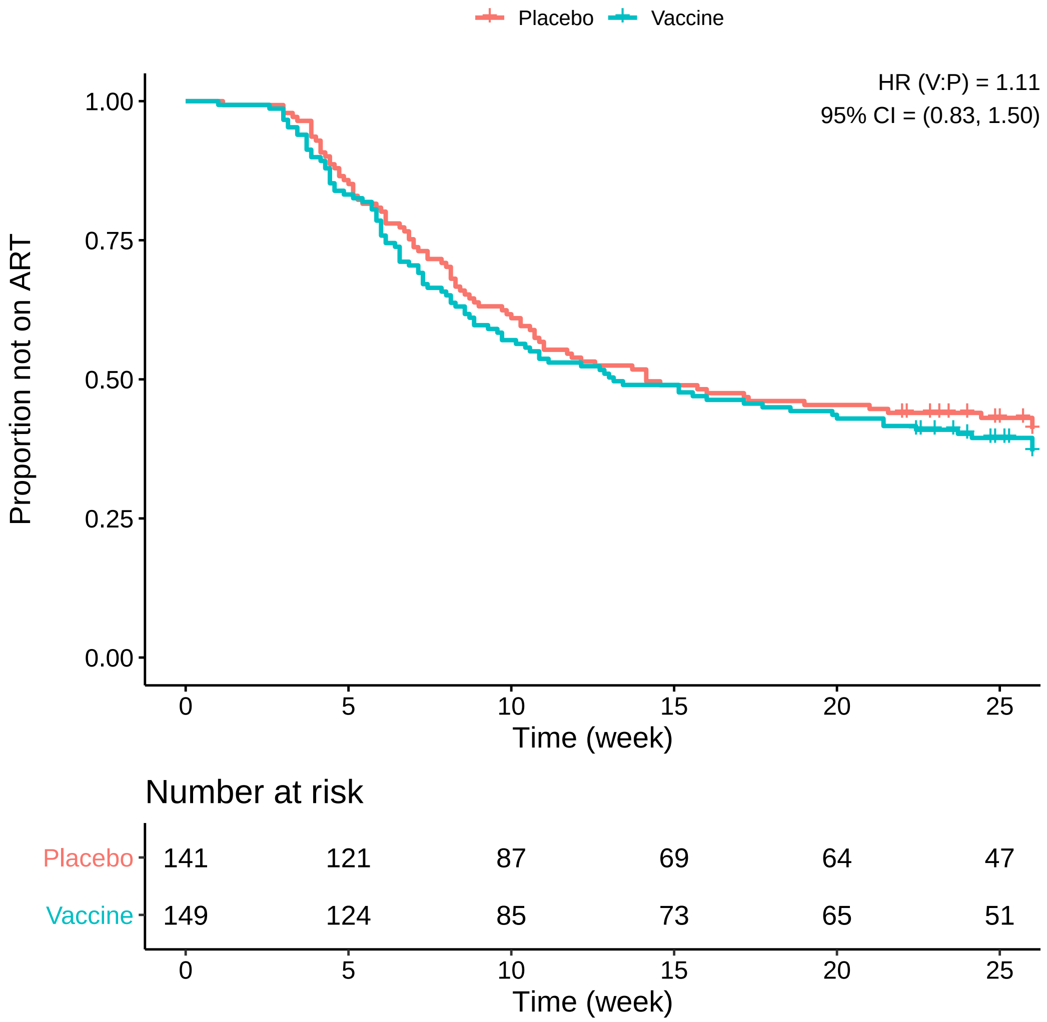

Figure S10: Kaplan-Meier plot for time to ART initiation among MITT infected participants. Log-rank test was used to compare event-time distributions between treatment groups.

2.10 Use of Pre-Exposure Prophylaxis (PrEP)

PrEP use was captured using self-report data at each visit, covering both PrEP and post-exposure prophylaxis (PEP) use, and using serial cross-sectional testing of a fixed number of participants. Specifically, dried blood spot (DBS) specimens were collected at each study site on the the 15th of every month for all participants visiting the clinic on that day. If the 15th of the month fell on a weekend or holiday, then specimens were collected on the nearest study day to it (either before or after). PrEP use was detected using a test that measures the amount of tenofovir-diphosphate (TFV-DP), a metabolite of tenofovir, in red blood cells. The test utilizes a dried blood spot as the sample source and analyzes for TFV-DP by liquid chromatography and tandem mass-spectroscopy, as previously described (Castillo-Mancilla et al. 2013; Bushman et al. 2011). Prevalence of TFV-DP was reported both as any detectable use and inferred effective use. A threshold of 16.6 fmol/sample was used to define detection of TFV-DP, a threshold of 1000 fmol/sample was used to define "effective" use for females at birth and 700 fmol/sample for males at birth.

Table S14: Number (%) of enrolled participants self-reporting PrEP or PEP use at any time during the study

	Female at Birth (N=3786)	Male at Birth (N=1618)	Total (N=5404)
PrEP Use	120 (3.2%)	52 (3.2%)	172 (3.2%)
PEP Use	91 (2.4%)	80 (4.9%)	171 (3.2%)

Table S15: PrEP/PEP use among enrolled participants by treatment group. Time period for self-reported usage is at the time of specimen collection.

<table>
<tr><th>Total Tested DBS Samples (N=2405)</th><th></th><th>Self-Report PrEP/PEP</th><th>Placebo Samples (N=1230)</th><th>Vaccine Samples (N=1175)</th></tr>
<tr><td rowspan="4">Detectable TFV-DP Level</td><td rowspan="2">Yes (N=51, 2.12%)</td><td>Yes (N=21)</td><td>12</td><td>9</td></tr>
<tr><td>No (N=30)</td><td>22</td><td>8</td></tr>
<tr><td rowspan="2">No (N=2354, 97.88%)</td><td>Yes (N=14)</td><td>7</td><td>7</td></tr>
<tr><td>No (N=2340)</td><td>1189</td><td>1151</td></tr>
<tr><td>Effective TFV-DP Level</td><td>Yes (N=5, 0.21%)</td><td>-</td><td>2</td><td>3</td></tr>
<tr><td>Estimated % person-years on Detectable PrEP/PEP</td><td></td><td></td><td>2.89%</td><td>1.99%</td></tr>
<tr><td>Estimated % person-years on Effective PrEP/PEP</td><td></td><td></td><td>0.27%</td><td>0.42%</td></tr>
</table>

Table S16: PrEP/PEP use among enrolled participants by sex at birth. Time period for self-reported usage is at the time of specimen collection.

Total Tested DBS Samples (N=2405)		Self-Report PrEP/PEP	Female at Birth Samples (N=1671)	Male at Birth Samples (N=734)
Detectable TFV-DP Level	Yes (N=51, 2.12%)	Yes (N=21)	12	9
		No (N=30)	20	10
	No (N=2354, 97.88%)	Yes (N=14)	10	4
		No (N=2340)	1629	711
Effective TFV-DP Level	Yes (N=5, 0.21%)	-	1	4
Estimated % person-years on Detectable PrEP/PEP			2.26%	2.87%
Estimated % person-years on Effective PrEP/PEP			0.18%	0.73%

2.11 Additional Safety Data and Analyses

Safety events are described by treatment received. Product administration errors were rare; 3 (0.11%) of vaccine group participants and 2 (0.07%) of placebo group participants received incorrect product at 1 visit, and no participants received incorrect product more than once.

The rates of four aggregate safety endpoints were compared between treatment groups, using Barnard's exact test. These aggregate endpoints are the rate of any reactogenicity, the rate of any related adverse event, the rate of any reactogenicity of grade 3 or higher, and the rate of any related adverse event of grade 3 or higher. All aggregate safety endpoints include events that occurred after any of the 6 vaccinations.

For the 18 deaths reported during the trial, causes of death comprised gunshot wound (3), stabbing (3), head trauma (1), motor vehicle accident (1), polytrauma (1), suicide (1), accidental electrocution (1), sudden death, presumably due to heart dysrhythmia or similar rare event (1), suspected pulmonary embolism (1), severe headache with chest pain and hemoptysis (1), drug-induced hepatic toxicity (1), mixed connective tissue disease (1), and tuberculosis (2), comprising 1 case of meningitis and 1 case of infectious granulomatous cerebral lesions.

Table S17: Local reactogenicities, systemic reactogenicities, and adverse events experienced by enrolled participants. Cells show the number (percent) of participants who experienced an event of a given severity across all vaccinations. Each participant was counted only once under the maximum severity of the events experienced.

		Vaccine (N=2706)	Placebo (N=2698)
Max. local reactogenicity			
	None	1915 (70.77%)	2469 (91.51%)
	Mild	555 (20.51%)	208 (7.71%)
	Moderate	198 (7.32%)	19 (0.70%)
	Severe	38 (1.40%)	2 (0.07%)
	Life threatening	0 (0.00%)	0 (0.00%)
	Any	791 (29.23%)	229 (8.49%)
	Any severe or greater	38 (1.40%)	2 (0.07%)
Max. systemic reactogenicity			
	None	1835 (67.81%)	1927 (71.42%)
	Mild	614 (22.69%)	540 (20.01%)
	Moderate	232 (8.57%)	210 (7.78%)
	Severe	24 (0.89%)	16 (0.59%)
	Life threatening	1 (0.04%)	5 (0.19%)
	Any	871 (32.19%)	771 (28.58%)
	Any severe or greater	25 (0.92%)	21 (0.78%)
Adverse events			
	None	977 (36.10%)	966 (35.80%)
	Mild	162 (5.99%)	137 (5.08%)
	Moderate	1445 (53.40%)	1470 (54.48%)
	Severe	98 (3.62%)	103 (3.82%)
	Life threatening	17 (0.63%)	12 (0.44%)
	Any	1730 (63.93%)	1732 (64.20%)
	Any severe or greater	123 (4.55%)	125 (4.63%)
	Death	8 (0.30%)	10 (0.37%)

Participants are counted once per reactogenicity or adverse event according the maximum severity level experienced across all vaccinations.

Table S18: Rates of aggregate safety endpoints by treatment received among enrolled participants, and the results of Barnard's test comparing event rates between treatment groups

	Vaccine		Placebo		
	%	(95% CI)	%	(95% CI)	Barnard's Test P-Value
Any reactogenicity	46.23	(44.36, 48.11)	32.84	(31.09, 34.63)	<0.001
Any related adverse event	1.40	(1.02, 1.92)	0.44	(0.25, 0.78)	<0.001
Any reactogenicity of grade 3 or higher	2.25	(1.76, 2.88)	0.89	(0.60, 1.32)	<0.001
Any related adverse event of grade 3 or higher	0.04	(0.00, 0.21)	0.00	(0.00, 0.14)	0.515

Table S19: Listing of adverse events of special interest (AESIs)[1] by treatment received (N=5404) among all enrolled participants

Trt	Counter	Publ. ID	Severity	Adverse Experience	Relation to Vaccine[2]	Num. Prev. Vacs.	Days Since Last Vacc.
Placebo	1	702-2771	Potentially life-threatening	Systemic lupus erythematosus	Not Related	4	371
	2	702-6677	Severe	Basedow's disease	Not Related	6	371
Vaccine	1	702-1974	Severe	Mass	Not Related	4	287
	2	702-4335	Moderate	Anti-neutrophil cytoplasmic antibody positive vasculitis	Not Related	5	152
	3	702-4991	Severe	Systemic lupus erythematosus	Not Related	2	602
	4	702-8809	Death	Mixed connective tissue disease	Not Related	5	355

[1] AEs of special interest (AESI) included, but were not limited to, potential immune-mediated diseases.

[2] Relationship of adverse event to study product is determined by site investigators.

Table S20: Grade 1-5 adverse events by system organ class, severity, and treatment received, ordered by decreasing frequency in safety cohort (N=5404)

System Organ Class / Severity	Total (N=5404)	Placebo (N=2698)	Vaccine (N=2706)
Participants with one or more AEs			
Mild and Greater	3459 (64.0%)	1732 (64.2%)	1727 (63.8%)
Moderate and Greater	3161 (58.5%)	1595 (59.1%)	1566 (57.9%)
Severe and Greater	247 (4.6%)	124 (4.6%)	123 (4.5%)
Life-threatening and Greater	47 (0.9%)	22 (0.8%)	25 (0.9%)
Fatal	18 (0.3%)	10 (0.4%)	8 (0.3%)
Infections and infestations			
Mild and Greater	2551 (47.2%)	1302 (48.3%)	1249 (46.2%)
Moderate and Greater	2450 (45.3%)	1254 (46.5%)	1196 (44.2%)
Severe and Greater	38 (0.7%)	20 (0.7%)	18 (0.7%)
Life-threatening and Greater	5 (0.1%)	5 (0.2%)	0 (0.0%)
Fatal	3 (0.1%)	3 (0.1%)	0 (0.0%)
General disorders and administration site conditions			
Mild and Greater	408 (7.5%)	199 (7.4%)	209 (7.7%)
Moderate and Greater	299 (5.5%)	149 (5.5%)	150 (5.5%)
Severe and Greater	6 (0.1%)	1 (0.0%)	5 (0.2%)
Life-threatening and Greater	6 (0.1%)	1 (0.0%)	5 (0.2%)
Fatal	2 (0.0%)	0 (0.0%)	2 (0.1%)
Reproductive system and breast disorders			
Mild and Greater	346 (6.4%)	178 (6.6%)	168 (6.2%)
Moderate and Greater	324 (6.0%)	166 (6.2%)	158 (5.8%)
Severe	4 (0.1%)	3 (0.1%)	1 (0.0%)
Nervous system disorders			
Mild and Greater	319 (5.9%)	151 (5.6%)	168 (6.2%)
Moderate and Greater	201 (3.7%)	95 (3.5%)	106 (3.9%)
Severe and Greater	8 (0.1%)	2 (0.1%)	6 (0.2%)
Life-threatening	2 (0.0%)	0 (0.0%)	2 (0.1%)
Gastrointestinal disorders			
Mild and Greater	318 (5.9%)	165 (6.1%)	153 (5.7%)
Moderate and Greater	204 (3.8%)	105 (3.9%)	99 (3.7%)
Severe and Greater	10 (0.2%)	6 (0.2%)	4 (0.1%)
Life-threatening	1 (0.0%)	1 (0.0%)	0 (0.0%)
Skin and subcutaneous tissue disorders			
Mild and Greater	271 (5.0%)	135 (5.0%)	136 (5.0%)
Moderate and Greater	186 (3.4%)	92 (3.4%)	94 (3.5%)
Severe	2 (0.0%)	1 (0.0%)	1 (0.0%)
Injury, poisoning and procedural complications			
Mild and Greater	269 (5.0%)	113 (4.2%)	156 (5.8%)
Moderate and Greater	219 (4.1%)	94 (3.5%)	125 (4.6%)
Severe and Greater	87 (1.6%)	41 (1.5%)	46 (1.7%)
Life-threatening and Greater	18 (0.3%)	8 (0.3%)	10 (0.4%)
Fatal	9 (0.2%)	6 (0.2%)	3 (0.1%)
Musculoskeletal and connective tissue disorders			
Mild and Greater	194 (3.6%)	99 (3.7%)	95 (3.5%)
Moderate and Greater	127 (2.4%)	60 (2.2%)	67 (2.5%)
Severe and Greater	8 (0.1%)	2 (0.1%)	6 (0.2%)
Life-threatening and Greater	2 (0.0%)	1 (0.0%)	1 (0.0%)
Fatal	1 (0.0%)	0 (0.0%)	1 (0.0%)

Table S20: Grade 1-5 adverse events by system organ class, severity, and treatment received, ordered by decreasing frequency in safety cohort (N=5404) *(continued)*

System Organ Class / Severity	Total (N=5404)	Placebo (N=2698)	Vaccine (N=2706)
Investigations			
Mild and Greater	187 (3.5%)	95 (3.5%)	92 (3.4%)
Moderate and Greater	63 (1.2%)	36 (1.3%)	27 (1.0%)
Severe	19 (0.4%)	14 (0.5%)	5 (0.2%)
Respiratory, thoracic and mediastinal disorders			
Mild and Greater	101 (1.9%)	43 (1.6%)	58 (2.1%)
Moderate and Greater	57 (1.1%)	24 (0.9%)	33 (1.2%)
Severe and Greater	8 (0.1%)	4 (0.1%)	4 (0.1%)
Life-threatening and Greater	3 (0.1%)	1 (0.0%)	2 (0.1%)
Fatal	1 (0.0%)	1 (0.0%)	0 (0.0%)
Psychiatric disorders			
Mild and Greater	56 (1.0%)	30 (1.1%)	26 (1.0%)
Moderate and Greater	49 (0.9%)	27 (1.0%)	22 (0.8%)
Severe and Greater	28 (0.5%)	16 (0.6%)	12 (0.4%)
Life-threatening and Greater	11 (0.2%)	5 (0.2%)	6 (0.2%)
Fatal	1 (0.0%)	0 (0.0%)	1 (0.0%)
Metabolism and nutrition disorders			
Mild and Greater	45 (0.8%)	20 (0.7%)	25 (0.9%)
Moderate and Greater	35 (0.6%)	14 (0.5%)	21 (0.8%)
Severe	10 (0.2%)	6 (0.2%)	4 (0.1%)
Vascular disorders			
Mild and Greater	45 (0.8%)	21 (0.8%)	24 (0.9%)
Moderate and Greater	26 (0.5%)	10 (0.4%)	16 (0.6%)
Severe and Greater	4 (0.1%)	1 (0.0%)	3 (0.1%)
Life-threatening	1 (0.0%)	0 (0.0%)	1 (0.0%)
Renal and urinary disorders			
Mild and Greater	44 (0.8%)	23 (0.9%)	21 (0.8%)
Moderate and Greater	19 (0.4%)	11 (0.4%)	8 (0.3%)
Severe	1 (0.0%)	0 (0.0%)	1 (0.0%)
Eye disorders			
Mild and Greater	33 (0.6%)	16 (0.6%)	17 (0.6%)
Moderate	22 (0.4%)	12 (0.4%)	10 (0.4%)
Neoplasms benign, malignant and unspecified (incl cysts and polyps)			
Mild and Greater	26 (0.5%)	14 (0.5%)	12 (0.4%)
Moderate and Greater	22 (0.4%)	11 (0.4%)	11 (0.4%)
Severe and Greater	2 (0.0%)	2 (0.1%)	0 (0.0%)
Life-threatening	1 (0.0%)	1 (0.0%)	0 (0.0%)
Blood and lymphatic system disorders			
Mild and Greater	22 (0.4%)	11 (0.4%)	11 (0.4%)
Moderate and Greater	14 (0.3%)	6 (0.2%)	8 (0.3%)
Severe	4 (0.1%)	3 (0.1%)	1 (0.0%)
Pregnancy, puerperium and perinatal conditions			
Mild and Greater	21 (0.4%)	12 (0.4%)	9 (0.3%)
Moderate and Greater	21 (0.4%)	12 (0.4%)	9 (0.3%)
Severe	21 (0.4%)	12 (0.4%)	9 (0.3%)
Ear and labyrinth disorders			
Mild and Greater	14 (0.3%)	3 (0.1%)	11 (0.4%)
Moderate and Greater	7 (0.1%)	1 (0.0%)	6 (0.2%)

Table S20: Grade 1-5 adverse events by system organ class, severity, and treatment received, ordered by decreasing frequency in safety cohort (N=5404) *(continued)*

System Organ Class / Severity	Total (N=5404)	Placebo (N=2698)	Vaccine (N=2706)
Severe	1 (0.0%)	0 (0.0%)	1 (0.0%)
Immune system disorders			
Mild and Greater	7 (0.1%)	3 (0.1%)	4 (0.1%)
Moderate	7 (0.1%)	3 (0.1%)	4 (0.1%)
Cardiac disorders			
Mild and Greater	6 (0.1%)	2 (0.1%)	4 (0.1%)
Moderate and Greater	3 (0.1%)	2 (0.1%)	1 (0.0%)
Severe and Greater	2 (0.0%)	1 (0.0%)	1 (0.0%)
Life-threatening	1 (0.0%)	0 (0.0%)	1 (0.0%)
Hepatobiliary disorders			
Mild and Greater	5 (0.1%)	0 (0.0%)	5 (0.2%)
Moderate and Greater	5 (0.1%)	0 (0.0%)	5 (0.2%)
Severe and Greater	4 (0.1%)	0 (0.0%)	4 (0.1%)
Life-threatening and Greater	2 (0.0%)	0 (0.0%)	2 (0.1%)
Fatal	1 (0.0%)	0 (0.0%)	1 (0.0%)
Social circumstances			
Mild and Greater	3 (0.1%)	2 (0.1%)	1 (0.0%)
Moderate	3 (0.1%)	2 (0.1%)	1 (0.0%)
Endocrine disorders			
Mild and Greater	2 (0.0%)	2 (0.1%)	0 (0.0%)
Moderate and Greater	2 (0.0%)	2 (0.1%)	0 (0.0%)
Severe	1 (0.0%)	1 (0.0%)	0 (0.0%)
Product issues			
Mild and Greater	1 (0.0%)	1 (0.0%)	0 (0.0%)
Moderate	1 (0.0%)	1 (0.0%)	0 (0.0%)

3 South African HIV-1 Sequence Data

Vaccine efficacy in RV144 was found to depend on HIV-1 viral genetics, especially the match of the exposing HIV-1 virus to the vaccine insert at position Env 169 in the V2 loop (Rolland et al. 2012). Figure S11 suggests that there may have been less matching of exposing viruses to the vaccine in HVTN 702 as compared to RV144 in the V1V2 region. We obtained two cohorts of HIV-1 Env protein sequences from the Los Alamos National Labs (LANL) HIV database (http://www.hiv.lanl.gov/): those sequences originating from Thailand and of any subtype, from the years of the RV144 study (2003-2006; n = 145 sequences), and those originating from South Africa and of any subtype, from the years of the HVTN 702 study, allowing for additional time to increase the sample size (2009-2018; n = 213 sequences). Sequences were compared to a given vaccine component, at each amino acid site in the V1V2 region of HIV-1 Envelope, and the proportion of sites with an amino acid residue matching that in the vaccine component was calculated over the V1V2 region. A lower proportion of match in V1V2 was observed for HVTN 702 vs. RV144, relative to both the ALVAC insert and to the vaccine proteins. In addition, whereas circulating viruses in Thailand were better matched to the RV144 vaccine than to the subtype 01_AE consensus sequence, South African viruses appear to be less well-matched to the HVTN 702 vaccine than to the subtype C consensus sequence.

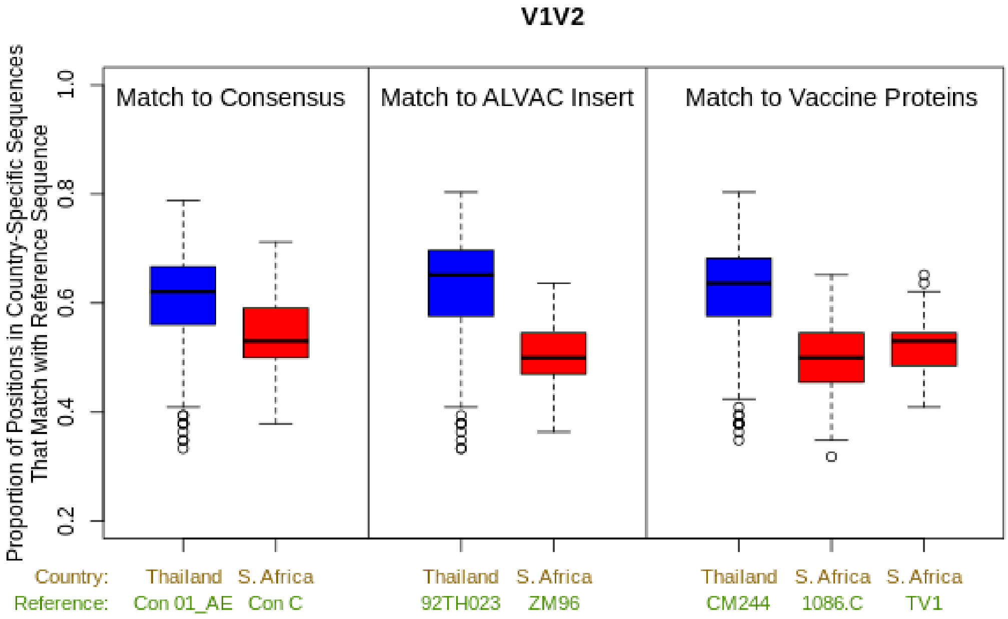

Figure S11: Proportion of amino acid sites in V1V2 where circulating HIV-1 sequences in South Africa vs. Thailand match the components of the HVTN 702 vs. RV144 HIV vaccines. Estimates are based on n = 213 sequences from South Africa, 2009-2017; and n = 145 sequences from Thailand, 2003-2006. Match frequency is shown for the ALVAC inserts, the vaccine proteins, and for the consensus sequences for South Africa and Thailand.

4 South African Host Genetics

Analyses in RV144 found evidence that the level of vaccine efficacy depended on host genetics, especially against HIV-1 viruses that were matched to the vaccine at position Env 169. Vaccine efficacy against 169-matched HIV-1's was estimated at 90.9% for participants with a Fc gamma receptor SNP rs114945036 C > T (CT/TT genotype; p = 0.001) as compared to an estimated vaccine efficacy of 15.2% (p = 0.52) for

other genotypes (Li et al. 2014). In RV144, there were 3 SNPs in perfect linkage disequilibrium, any of which could be the causal variant; the table below shows the frequencies of each in the RV144 data (n = 205 participants genotyped). Also shown in the table are frequencies of each of these FCR genotypes in South Africans, based on data from Lassauniere and Tiemessen (2016) (n = 131 South African Blacks). One of the 3 SNPS had a higher prevalence than in RV144, but the other two were not observed at all. Overall, the 3-variant haplotype was not observed in South Africa.

Table S21: Low prevalence of 2 linked Fc gamma receptor SNPs in South African population

CRF host genotype found to modify VE	Prevalence, RV144 (n = 205)	Prevalence, South African Blacks (n = 131)
rs114945036 C > T	14% minor allele freq.	25% minor allele freq.
	24% for CT/TT	49% for CT/TT
rs138747765 C > T	14% minor allele freq.	0% minor allele freq.
	24% for CT/TT	0% for CT/TT
rs78603008 G > A	14% minor allele freq.	0% minor allele freq.
	24% for GA/AA	0% for GA/AA

RV144 analyses also found evidence that VE was higher among individuals carrying an HLA A*02 allele, and again the result was most strong against 169-matched viruses. The estimated VE against 169-matched viruses was 74% for those carrying A*02 vs 15% for those not carrying A*02 (p = 0.01 for interaction test) (Gartland et al. 2014). Whereas the A*02 allele was frequent in the RV144 study (50% prevalence, n = 450 Thai participants with HLA data) (Prentice et al. 2014), it appears to be much less frequent among South African Blacks, based on data from the HVTN 503 Phambili HIV vaccine trial (26% prevalence, n = 322 South Africans) (Hertz et al. 2016) and a recent review of HLA diversity in southern Africa (Tshabalala, Mellet, and Pepper 2015).

References

Benkeser, DC, M Carone, and PB Gilbert. 2017. "Improved Estimation of the Cumulative Incidence of Rare Outcomes." *Statistics in Medicine*. Wiley-Blackwell. https://doi.org/10.1002/sim.7337.

Benkeser, DC, PB Gilbert, and M Carone. 2019. "Estimating and Testing Vaccine Sieve Effects Using Machine Learning." *Journal of the American Statistical Association* 114 (527). Taylor & Francis: 1038–49.

Benkeser, DC, and NS Hejazi. 2017. *survtmle: Targeted Minimum Loss-Based Estimation for Survival Analysis in R*. https://github.com/benkeser/survtmle. https://doi.org/10.5281/zenodo.835868.

Bushman, LR, JJ Kiser, JE Rower, B Klein, JH Zheng, ML Ray, and PL Anderson. 2011. "Determination of Nucleoside Analog Mono-, Di-, and Tri-Phosphates in Cellular Matrix by Solid Phase Extraction and Ultra-Sensitive Lc-Ms/Ms Detection." *J Pharm Biomed Anal* 56: 390–401.

Castillo-Mancilla, JR, JH Zheng, JE Rower, A Meditz, EM Gardner, J Predhomme, C Fernandez, et al. 2013. "Tenofovir, Emtricitabine, and Tenofovir Diphosphate in Dried Blood Spots for Determining Recent and Cumulative Drug Exposure." *AIDS Res Hum Retroviruses* 29: 384–90.

Gartland, AJ, SS Li, J McNevin, GD Tomaras, R Gottardo, H Janes, Y Fong, et al. 2014. "Analysis of Hla a*02 Association with Vaccine Efficacy in the Rv144 Hiv-1 Vaccine Trial." *J Virol.* 88: 8242–55.

Hertz, T, MG Logan, M Rolland, CA Magaret, C Rademeyer, A Fiore-Gartland, PT Edlefsen, et al. 2016. "A Study of Vaccine-Induced Immune Pressure on Breakthrough Infections in the Phambili Phase 2b Hiv-1 Vaccine Efficacy Trial." *Vaccine* 34: 5792–5801.

Lassauniere, R, and CT Tiemessen. 2016. "Variability at the Fcgr Locus: Characterization in Black South Africans and Evidence for Ethnic Variation in and Out of Africa." *Genes Immun.* 17: 93–104.

Li, SS, PB Gilbert, GD Tomaras, G Kijak, G Ferrari, R Thomas, CW Pyo, et al. 2014. "FCGR2C Polymorphisms Associate with Hiv-1 Vaccine Protection in Rv144 Trial." *The Journal of Clinical Investigation* 124: 3879–90.

Prentice, HA, PK Ehrenberg, KM Baldwin, A Geretz, C Andrews, S Nitayaphan, S Rerks-Ngarm, et al. 2014. "HLA Class I, Kir, and Genome-Wide Snp Diversity in the Rv144 Thai Phase 3 Hiv Vaccine Clinical Trial." *Immunogenetics* 66: 299–310.

Rolland, M, PT Edlefsen, BB Larsen, S Tovanabutra, E Sanders-Buell, T Hertz, AC deCamp, et al. 2012. "Increased Hiv-1 Vaccine Efficacy Against Viruses with Genetic Signatures in Env V2." *Nature* 490: 417–20.

Tshabalala, M, J Mellet, and MS Pepper. 2015. "Human Leukocyte Antigen Diversity: A Southern African Perspective." *Journal of Immunology Research*.
